# Intercalation of Transition Metals into MXenes: Impact on Electronic and Pseudocapacitive Properties

**DOI:** 10.1021/acsnano.5c06170

**Published:** 2025-09-05

**Authors:** Shianlin Wee, Xiliang Lian, Dario Gomez Vazquez, Mathieu Salanne, Maria R. Lukatskaya

**Affiliations:** † Electrochemical Energy Systems Laboratory, Department of Mechanical and Process Engineering, ETH Zurich, 8092 Zurich, Switzerland; ‡ 27063 Sorbonne Université, CNRS, Physicochimie des Électrolytes et Nanosystèmes Interfaciaux, F-75005 Paris, France; § Réseau sur le Stockage Electrochimique de l’Energie (RS2E), FR CNRS 3459, 80039 Amiens Cedex, France; ∥ Institut Universitaire de France (IUF), 75231 Paris, France

**Keywords:** 2D materials, MXenes, transition metal intercalation, ab initio molecular dynamics, density functional theory, pseudocapacitance, charge storage mechanism, in situ X-ray absorption spectroscopy

## Abstract

MXenes are two-dimensional transition metal carbides and nitrides characterized by versatile electronic and electrochemical properties. Herein, we investigate the electronic interactions between various redox-active transition metals (Ni, Co, Mn, and Zn) intercalated into the conductive Ti_3_C_2_T_*x*_ MXene host. Employing X-ray absorption spectroscopy (XAS) and Bader charge analysis, we reveal that the oxidation states of the intercalated ions remain unchanged upon insertion, whereas Ti atoms within the MXene layers become progressively oxidized with increasing intercalant concentration. Consequently, the electrical resistivity of the intercalated MXenes increases. Ab initio molecular dynamics (AIMD) and density functional theory (DFT) demonstrate distinct spatial arrangements and coordination environments of the intercalated cations, significantly influencing their electronic density of states and interactions with MXene surfaces. Pseudocapacitance measurements in 0.1 M NaOH show distinct behaviors: Co exhibits significant redox activity with less participation from Ti of MXene, while Ni ions show negligible oxidation state changes with predominant Ti redox involvement. Our findings reveal the complex electronic and redox behavior of transition metal-intercalated MXenes, guiding the targeted modification of 2D material properties through careful selection of intercalant species.

## Graphical abstract

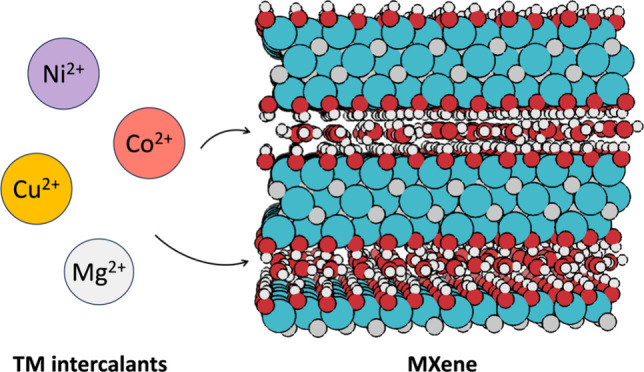

## Introduction

MXenes are an emerging class of 2D transition metal carbides and nitrides with the general formula M_*n*+1_X_*n*_T_*x*_. Here, M represents an early transition metal, *n* = 1–4, X is carbon and/or nitrogen, and T_*x*_ refers to surface terminations such as =O, −OH, −Cl, and −F.^1^ By combining tunable metallicity,^1^ hydrophilicity,^1^ chemical tunability,^1^ and attractive redox characteristics,^2^ MXenes show a great promise for diverse applications spanning from energy storage,^2–4^ electrocatalysis,^5^ and electromagnetic shielding^6^ to biomedical uses.^7^ The electronic and electrochemical properties of MXenes are highly tunable through their composition,^1^ structure,^1,8^ and surface termination.^8^ Furthermore, the layered structure and negatively charged 2D surfaces of MXenes facilitate (electro)­chemical intercalation of various cations^4,9,10^ and polar molecules,^11^ offering an additional route to alter their properties.

Despite extensive research on the intercalation of alkali,^4^ alkaline earth,^4^ and organic cations into MXenes, very few studies have focused on the intercalation of TM cations. Most of these works focus on anchoring single atoms or nanoparticles of transition metals (TMs) onto MXene surfaces.^12–16^ Therefore, the interfacial interactions and chemistries between TM cations and MXenes within the confined environment between MXene layers remain relatively unexplored. Our prior research shows that intercalation of Cu ions into Ti_3_C_2_T_*x*_ induces charge redistribution within MXene layers, leading to a unique redox reaction of the guest Cu ions upon intercalation and altering their electronic and electrochemical properties.^17^

In this work, we investigate the impact of intercalating redox-active 3d transition metal cations (Mn^2+^, Co^2+^, Ni^2+^, Cu^2+^, and Zn^2+^) into the Ti_3_C_2_T_*x*_ MXene host. These TMs were selected due to their comparable electronic configurations, enabling a systematic comparison with our earlier findings. Moreover, the intercalation of 4d and 5d TMs into MXenes presents significant synthetic challenges, as these metals tend to undergo reduction prior to successful incorporation between the MXene layers, often leading to undesired phase formation. Therefore, the use of 3d TMs provides both chemical compatibility and experimental feasibility for exploring interlayer interactions and redox behavior. By systematically varying the TM, we elucidate their influence on the MXene’s electronic and electrochemical properties. A combination of ab initio molecular dynamics (AIMD), density functional theory (DFT), X-ray absorption spectroscopy (XAS), resistivity, and electrochemical measurements was employed to comprehensively characterize the resulting physicochemical changes. Finally, using in situ XAS experiments to monitor the oxidation state changes of TM-intercalated MXenes under applied potential, we reveal that the electrochemical response is strongly influenced by the intercalated TM cation.

## Results and Discussion

### Electronic Structure of TM-Intercalated Ti_3_C_2_T_*x*_

First, we perform ab initio molecular dynamics (AIMD) calculations for a detailed comparison of how the electronic structure of pristine Ti_3_C_2_T_*x*_ MXene changes upon intercalation of identical fractions of 0.2 Ni^2+^, Co^2+^, Cu^2+^, and Mg^2+^ cations per Ti_3_C_2_(OH)_2_ unit.^17^ To provide a redox-silent baseline, we included closed-shell Mg^2+^ in the AIMD set. This electrochemically inert cation isolates purely structural effects—interlayer expansion and solvation—from the redox-coupled electronic contributions of 3d transition-metal guests. The choice of AIMD was motivated by (1) the occurrence of chemical reactions, such as proton transfer, within the materials, that cannot be easily included in classical MD and (2) the limitations of current force fields in accurately modeling transition metals due to their complex interaction with water and MXenes.^18^ A systematic approach, detailed in the Materials and Methods section, was employed to identify representative configurations. The final compositions of the simulation cells are summarized in Table S1.

Figure 1 shows typical snapshots of the simulated MXene structures with different intercalated ions. Upon intercalation, the water molecules adopt a bilayer-like structure with increasing interlayer spacing. This reorganization is driven by the hydration of the cations in the interlayer. Notably, we observe distinct spatial arrangements of the cations within the interlayer for each intercalating cation: Co^2+^ and Mg^2+^ cations reside within the water bilayer, without direct contact with the MXene termination groups, whereas Cu^+^ and Ni^2+^ are predominantly coordinated by water oxygen atoms and MXene surface terminations.

**Figure 1:**
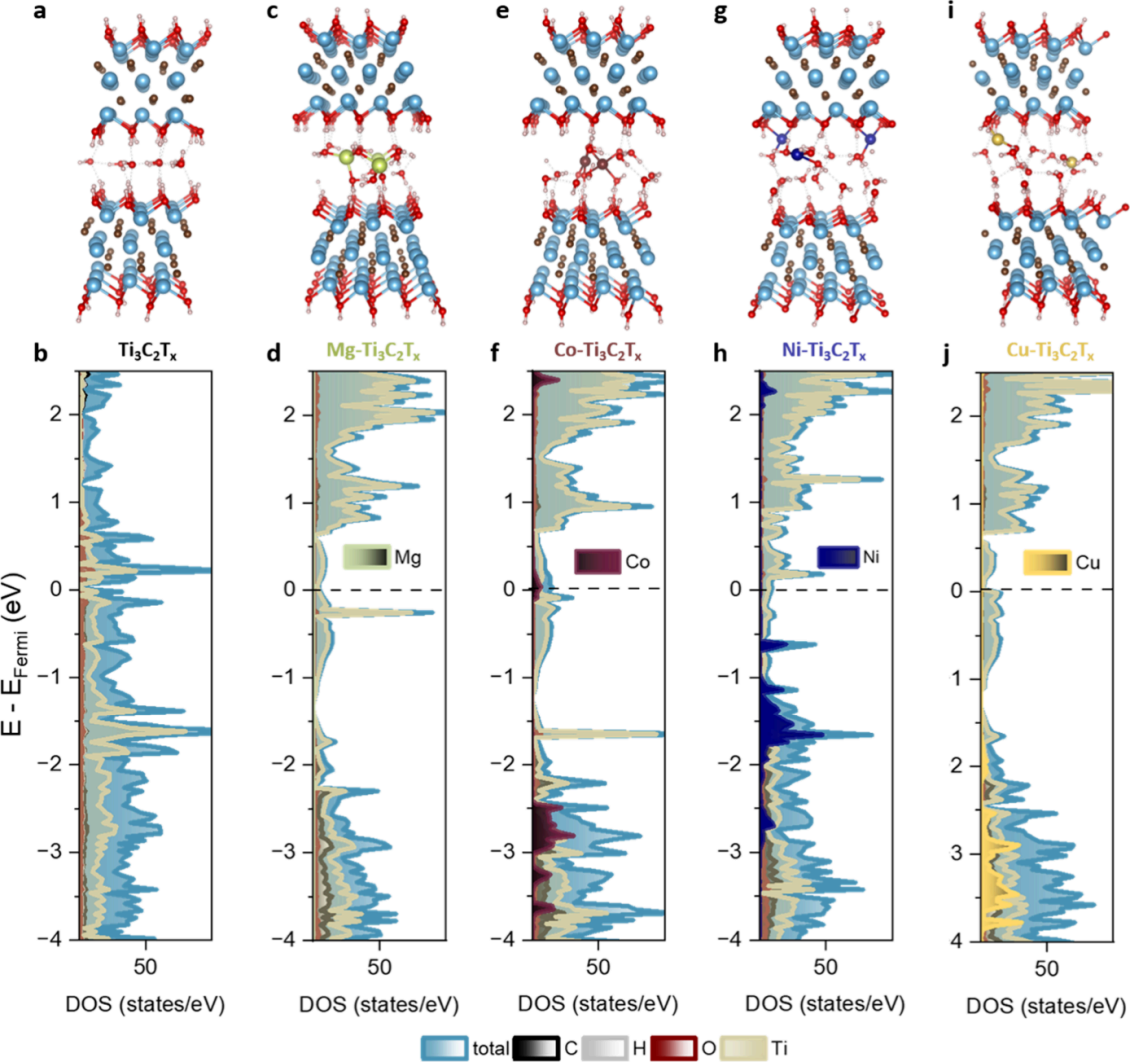
Top panels: typical snapshots extracted from the simulations of (a) pristine MXene with a single-layer H_2_O, as well as MXenes inserted with a 0.2 fraction of intercalated cations including (c) Mg, (e) Co, (g) Ni, and (i) Cu. The Ti, C, O, and H atoms are displayed in cyan, brown, red, and white spheres, respectively. The metal cations in between the layers are shown in green, wine, blue, and yellow for Mg, Co, Ni, and Cu, respectively. Albeit two MXene layers are shown for visualization purposes, the DFT model only includes one layer of MXene between two layers of water to mimic the experimental situation. Bottom panels: the corresponding density of states for (b) pristine MXenes and (d) Mg, (f) Co, (h) Ni, and (j) Cu-inserted MXenes.

To further understand these structural differences, we calculated the radial distribution functions (RDF) and coordination numbers (CNs) between cations and oxygen atoms, as shown in Figure S1. Table S2 summarizes the extracted average first-neighbor distances and first solvation shell CNs. For all the transition metal cations (TM), the first TM-O distance remains comparable to the bulk solution values, consistent with prior studies on MXenes intercalated with alkali and alkali-earth metal cations.^19^ Meanwhile, the CNs of the studied intercalated cations exhibit distinct behaviors compared to those of bulk solution. Previous studies have shown that alkali and alkali-earth cations experience reduced CNs when confined in interlayer spaces.^19^ Our findings for Mg^2+^ align with these observations, as its CN decreases from 6 in bulk water to 4 in the interlayer environment. For transition metal cations, we see that CNs also decrease upon intercalation. For Cu cations, consistent with our previous findings,^17^ the CN can drop as low as 2 due to the reduction of Cu^2+^ to Cu^+^ upon intercalation. Meanwhile, intercalated Ni^2+^ and Co^2+^ are coordinated by 3 and 4 oxygen atoms, respectively, compared to CN = 6 in bulk solution.^20,21^ This suggests that their electronic properties may differ significantly from those of Cu-MXene. Additionally, the coordination shell of Co^2+^ is more well-defined compared to that of Ni^2+^, as indicated by the presence of a plateau for Co^2+^ in the CN­(r) function (Figure S1). This distinction likely arises from their different positions within the interlayer (Figure 1).

Next, we calculated the Bader charges for the intercalated TM cations (Table S3) and compared them to those of the corresponding TM oxides. Upon intercalation, Co ions retain a +2 oxidation state, as indicated by its Bader charge of 0.9520, which is close to the value of 1.1650 in CoO. The case of Ni is more complex. The Ni Bader charge in NiO is 1.130, whereas we obtain a value of 0.7191 for Ni-MXene. Although this might suggest a partial reduction of Ni^2+^ upon intercalation, caution is necessary due to the absence of a reliable reference value for Ni^1+^. We propose that the Ni–O bonds formed with the oxygen atoms of the MXene surface, particularly those bound to oxidized Ti atoms, might induce charge accumulation on the Ni atoms, leading to an artificially low Bader charge (see Figure S2 for an illustration of the effect of bonding on the charge density around the Ni atoms). To verify this hypothesis, we performed another calculation where we substituted the Co cations in Co-MXene with Ni cations and then calculated the Bader charges. The resulting Ni average Bader charge is 0.9718, close to that of NiO. This result confirms that the lower Bader charge observed in the initial calculations is likely an artifact of the Bader charge analysis algorithm.

Finally, we analyzed the total density of states (TDOS) and partial density of states (PDOS) of different TM-intercalated MXenes. We calculated the DOS using the hybrid functional HSE06 (Figure 1) and PBE (Figure S3) and found subtle differences. In general, the DOS contributions from Ti, C, and H atoms remain similar to those of pristine MXene. For all cases examined, the DOS at the Fermi level is dominated by Ti contributions, consistent with the metallic nature of the MXenes. As expected, no Mg-related states were observed in the displayed energy range due to its closed-shell electron configuration. In contrast, the 3d orbitals of Co^2+^ and Ni^2+^ contribute notably to the DOS near and below the Fermi level. However, in the case of Cu-MXene, Cu-related DOS contributions are absent at the Fermi level. These important differences underscore the unique ways in which each transition metal influences the DOS of intercalated MXene. We note here that we did not pursue AIMD studies of Mn^2+^ and Zn^2+^ because the highest achievable experimental loading (discussed below) for these cations is only ∼0.09 ion per Ti_3_C_2_T_*x*_—effectively one ion per supercell, making the setup inconsistent with our other TM simulations, where higher loadings correspond to two cations.

### Characterization of TM-Intercalated Ti_3_C_2_T_*x*_

Simulations revealed that different TM cations result in distinct electronic structures when intercalated into MXenes. Therefore, to validate our simulation results experimentally, we synthesized Ni-, Co-, Zn-, and Mn-intercalated Ti_3_C_2_T_*x*_ MXenes. The X-ray diffraction (XRD) patterns (Figure S4) revealed a shift of the (002) peak from 2θ = 7.9 ° (*d*-spacing = 11.2 Å) for pristine MXene to approximately 2θ = 5.8 ° (*d*-spacing = 15.2 Å) for TM-intercalated MXene samples, confirming the successful insertion of TM cations. Additionally, no TM or oxide peaks were observed, further indicating that the TM cations remained in their ionic form after intercalating between MXene layers.

To quantify the TM content, we employed inductively coupled plasma–optical emission spectrometry (ICP–OES) and scanning electron microscopy (SEM)–energy dispersive X-ray spectroscopy (EDX). The resulting fractions of TMs per unit of Ti_3_C_2_T_*x*_ were confirmed as follows: Ni_0.09 ± 0.003_, Ni_0.13 ± 0.005_, Ni_0.31 ± 0.012_, Co_0.09 ± 0_, Co_0.20 ± 0.005_, Zn_0.09 ± 0_, and Mn_0.09 ± 0.008_ (Figure S5). The fractions of Zn and Mn cations remained rather low (around 0.09 per unit of Ti_3_C_2_T_*x*_), regardless of the Zn^2+^ or Mn^2+^ concentration of the intercalation solution. These observations highlight that the fraction of intercalated TM depends not only on the concentration of the intercalant solution and the interaction between TM cations and MXenes but also on the nature of the TM itself.^17^ Finally, scanning transmission electron microscope (STEM)–energy dispersive X-ray spectroscopy (EDX) maps show the homogeneous distribution of the TMs within Ti_3_C_2_T_*x*_ layers and the absence of TM nanoparticles on the MXene surface (Figures S6–S12).

Next, using XAS and conductivity measurements, we investigated the effects of TMs intercalants on the properties of MXenes. For all studied samples, the Ti K-edges shifted to higher energy compared to the pristine ones, indicating that Ti atoms partially oxidize upon the insertion of guest TM intercalants (Co, Ni, Mn, and Zn ions) (Figure 2a). This observation aligns with our previous findings, where upon intercalation of Cu ions, partial oxidation of Ti atoms takes place.^17^

**Figure 2:**
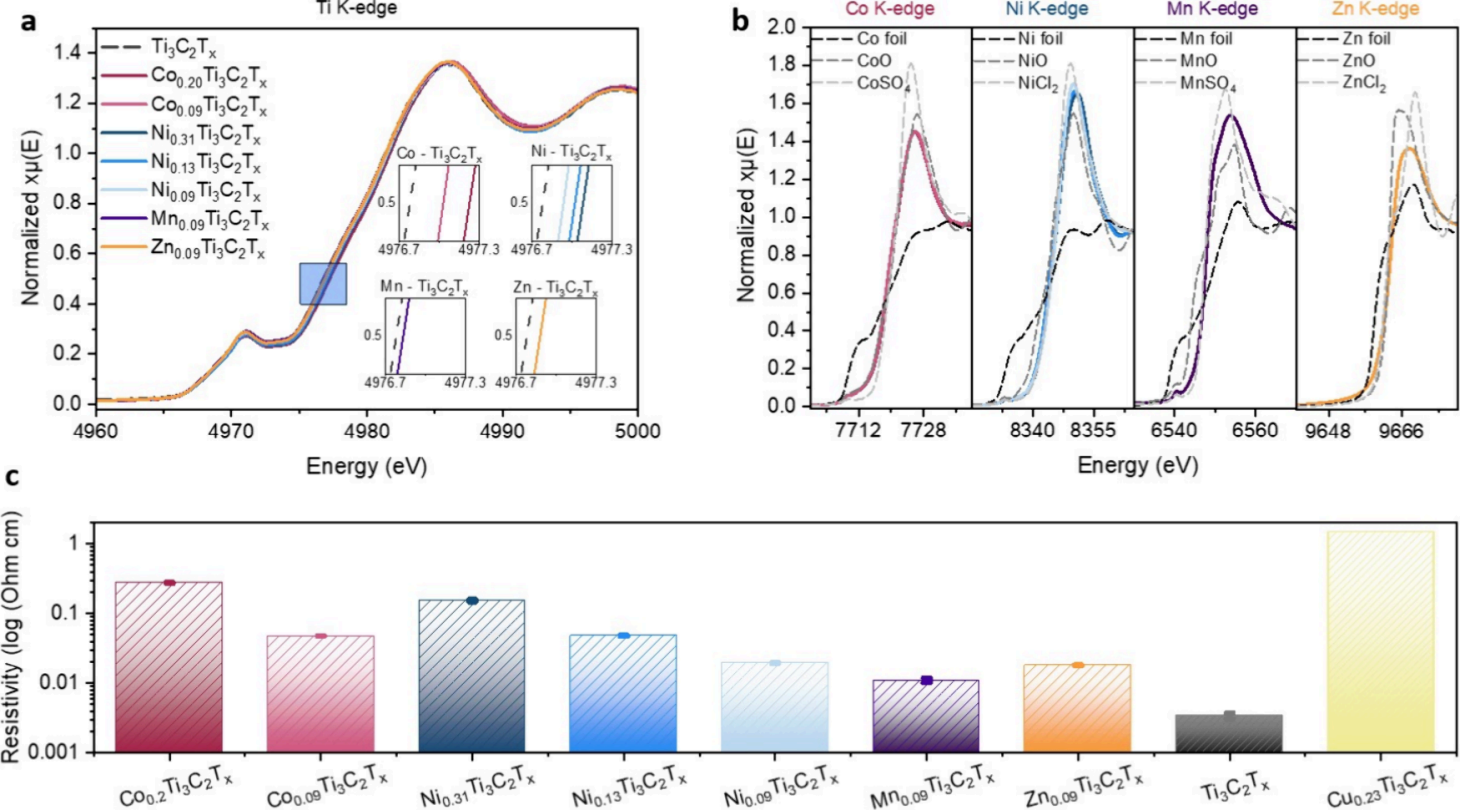
(a) Normalized X-ray absorption spectra at the Ti K-edge for Ti_3_C_2_T_*x*_, Ni–Ti_3_C_2_T_*x*_, Co–Ti_3_C_2_T_*x*_, Zn–Ti_3_C_2_T_x_, and Mn–Ti_3_C_2_T_*x*_. (b) Normalized X-ray absorption spectra of Co–Ti_3_C_2_T_*x*_, Ni–Ti_3_C_2_T_*x*_, Mn–Ti_3_C_2_T_*x*_, and Zn–Ti_3_C_2_T_*x*_ at their respective inserted transition metal’s K-edge. (c) Resistivity measurements of pristine and intercalated Ti_3_C_2_T_*x*_.

To further explore the relationship between the Ti oxidation state and the electrical conductivity of the MXene host, we investigated the influence of (1) different transition metal (TM) intercalants (Co, Ni, Mn, and Zn) and (2) varying TM intercalant fractions. Our results show that for the same TM, increasing the TM intercalant fraction leads to a higher Ti oxidation state (Figure 2a). The trend is accompanied by a decrease in electrical conductivity, as evidenced by the lower conductivity of Ni_0.31_Ti_3_C_2_T_*x*_, compared to those of Ni_0.13_Ti_3_C_2_T_*x*_ and Ni_0.09_Ti_3_C_2_T_*x*_, and similarly for Co_0.20_Ti_3_C_2_T_*x*_, compared to Co_0.09_Ti_3_C_2_T_*x*_ samples (Figure 2c).

Next, we compared Ti_3_C_2_T_*x*_ samples intercalated with similar fractions of various TMs (Mn, Zn, Ni, and Co). An increase in the Ti K-edge energy, corresponding to a higher Ti oxidation state, followed this sequence: Mn_0.09_Ti_3_C_2_T_*x*_, Zn_0.09_Ti_3_C_2_T_*x*_, Ni_0.09_Ti_3_C_2_T_*x*_, and Co_0.09_Ti_3_C_2_T_*x*_ (Figure 2a). Notably, this trend is mirrored by a corresponding increase in sample resistivity (Figure 2c). Finally, we compared the conductivity of MXenes with different types of TM and varying fractions to provide an overview of the effects of TM types and fractions. The following trend in electrical conductivity was observed: Ti_3_C_2_T_*x*_ > Mn_0.09_Ti_3_C_2_T_*x*_ > Zn_0.09_Ti_3_C_2_T_*x*_ > Ni_0.09_Ti_3_C_2_T_*x*_ > Co_0.09_Ti_3_C_2_T_*x*_ ∼ Ni_0.13_Ti_3_C_2_T_*x*_ > Ni_0.31_Ti_3_C_2_T_*x*_ > Co_0.20_Ti_3_C_2_T_*x*_. Simultaneously, the oxidation states of Ti increase in the same sequence (Figure 2c). These results suggest that increased oxidation state of Ti MXene can lead to a decrease in charge carrier density near the Fermi level, and consequently, result in increased resistivity in TM-intercalated Ti_3_C_2_T_*x*_.^17^ Nonetheless, it can be seen that the Cu_0.23_Ti_3_C_2_T_*x*_ displayed higher resistivity compared to all other TM-intercalated MXene, despite its Ti K-edge has lesser shift compared to one of Co_0.20_Ti_3_C_2_T_*x*_ (Figure S13). This can be attributed to the different nature and resulting DOS for Cu^2+^ when comparing with Co^2+^ and Ni^2+^.

Next, we performed XAS at the respective TM K-edge to probe the chemical state of intercalated TM cations. XAS revealed that Ni, Co, Mn, and Zn cations maintain a +2 oxidation state upon intercalation (Figure 2b). This is in contrast to the case of Cu cation intercalation into MXene, which results in charge redistribution and partial reduction of Cu^2+^ to approximately +1.3. This difference can potentially be attributed to the electronic configuration of the Cu^2+^ ion ([Ar]­3d^9^4s^0^), which is lacking 1e^–^ electron to complete its 3d shell. As the 3d^10^ configuration is more energetically favorable, upon insertion into the highly conductive Ti_3_C_2_T_*x*_ MXene host, electron density shifts toward the Cu ion guests. This is not the case for the other studied TM cations such as Ni^2+^ ([Ar]­3d^8^4s^0^), Co^2+^ ([Ar]­3d^7^4s^0^), Mn^2+^ ([Ar]­3d^5^4s^0^), and Zn^2+^ ([Ar]­3d^10^4s^0^); as a result, they maintain a valency of +2.

### Charge Storage Mechanism in TM-Intercalated Ti_3_C_2_T_*x*_

We can therefore expect a distinct electrochemical response for each TM-intercalated MXene. Given their higher Co and Ni content, Co_0.20_Ti_3_C_2_T_*x*_ and Ni_0.31_Ti_3_C_2_T_*x*_ MXenes were selected for further in-depth electrochemical studies using in situ XAS to accurately track changes in Co and Ni XAS and quantify their contributions to charge storage. Interestingly, cyclic voltammetry (CV) profiles of Co_0.20_Ti_3_C_2_T_*x*_ show no discernible redox peaks compared to those of the pristine MXene (scan rate of 1 mVs^–1^ in 0.1 M NaOH, Figures 3a and S14). This contrasts with the case of Cu-intercalated Ti_3_C_2_T_*x*_, for which the distinct redox peaks were observed in CVs,^17^ while in situ XAS confirmed the redox activity of Cu ions and their contribution to the overall pseudocapacitance of Cu–Ti_3_C_2_T_*x*_ alongside the Ti of the MXene host.^17^

**Figure 3:**
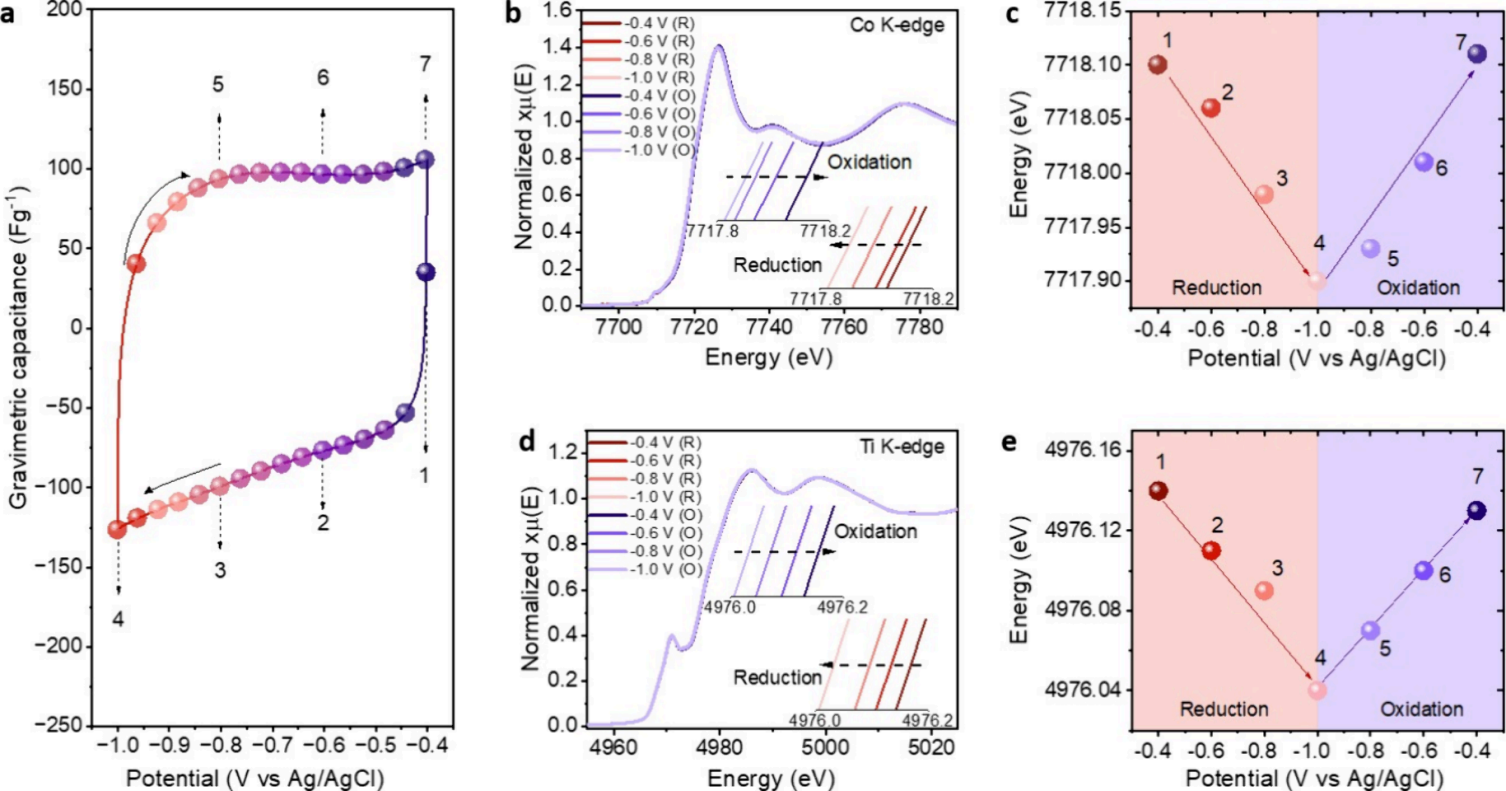
(a) CV of Co_0.20_Ti_3_C_2_T_*x*_ at 1 mVs^–1^. In situ XAS data of Co_0.20_Ti_3_C_2_T_*x*_. (b) Co K-edge XANES spectra collected at different applied potentials and (c) variation of Co edge energy with applied potential. (d) Ti K-edge XANES spectra at various reduction and oxidation potentials and (e) changes in Ti edge energy versus potential during negative and positive potential sweeps.

Next, to elucidate the role of Co ions in the charge storage mechanism of Co_0.20_Ti_3_C_2_T_*x*_, in situ XAS was employed to investigate the redox changes in both intercalated Co ions and Ti atoms of the MXene structure. The linear decrease in Co K-edge energy during the cathodic scan, followed by its recovery to the initial position during the anodic scan (Figures 3b,c and S15, S16), indicates reversible redox reactions of the intercalated Co cations. These findings strongly suggest the participation of Co ions in the pseudocapacitive charge storage.

To quantitatively estimate the contributions of Co redox to the overall Co–Ti_3_C_2_T_*x*_ capacitance, we correlated Co edge energy shifts to its valency. We can infer that each Co ion gains approximately 0.2 *e* ^–^ during charging within the voltage window of 0.6 V. The Ti K-edge energy, as expected, showed a similar trend (Figures 3d,e and S17), revealing an average gain of 0.08 *e* ^–^ per each Ti_3_C_2_T_*x*_ due to partial reduction of Ti within the MXene structure during charging. Considering the contributions of both Co and Ti redox, the total electron gain for each Co_0.20_Ti_3_C_2_T_*x*_ unit is 0.12 *e* ^–^, which can be correlated to a redox capacitance of 90 F g^–1^. This value is in excellent agreement with the overall capacitance of 93 F g^–1^ calculated from electrochemistry measurements, confirming the combined contribution of intercalated Co ions and the Ti MXene host redox to charge storage. Importantly, despite distinct differences in their CV profiles, both Co–Ti_3_C_2_T_*x*_ (this study) and Cu–Ti_3_C_2_T_*x*_ ^17^ exhibit unique guest–host pseudocapacitance. Moreover, the Co_0.20_Ti_3_C_2_T_*x*_ material demonstrated excellent cycling stability, maintaining 99.5% of its capacitance after 10,000 cycles in 0.1 M NaOH electrolyte (Figures S18–S20). Also, we performed EXAFS analysis of Co during charging/discharging processes (Figure S21), which revealed minimal changes in the coordination environment of Co ions. This suggests that the intercalated ions preserve their local environment throughout cycling without experiencing irreversible oxidation or migration out of the interlayers.

Given the difference in CV profiles between Cu–Ti_3_C_2_T_*x*_ (distinct redox CV peak) and Co–Ti_3_C_2_T_*x*_ (square-shaped CV), fundamental questions arise: does the absence of redox peaks associated with changes in the oxidation state in Co_0.20_Ti_3_C_2_T_*x*_ represent a special case? Can general trends be identified for different TM-Ti_3_C_2_T_*x*_ samples? To elucidate this, we used in situ XAS to study the charge storage mechanism of Ni_0.31_Ti_3_C_2_T_*x*_ as its CV profiles have no pronounced redox peaks compared to pristine Ti_3_C_2_T_*x*_, similar to Co_0.20_Ti_3_C_2_T_*x*_ (Figures 4a and S22). Interestingly, no notable changes in the Ni K-edge energy were observed for Ni_0.31_Ti_3_C_2_T_*x*_ samples during charge/discharge (Figures 4b,c and S23). Meanwhile, the Ti K-edge energy decreased linearly during charging (Figures 4d,e and S24); therefore, we can quantify that each Ti_3_C_2_T_*x*_ gains 0.18 *e* ^–^ during charging, which can be calculated as a redox capacitance value of 98 F g^–1^. This value is very close to 102 F g^–1^ that we obtained from electrochemical measurements. These observations suggest that intercalated Ni ions do not exhibit redox activity and do not contribute to pseudocapacitance after being introduced between the MXene layers, and only Ti of Ti_3_C_2_T_*x*_ actively participates in the charge storage of Ni_0.31_Ti_3_C_2_T_*x*_. Similar to Co_0.20_Ti_3_C_2_T_*x*_, Ni_0.31_Ti_3_C_2_T_*x*_ demonstrated excellent cycling stability, maintaining 99% of its capacitance and TM loading after 10,000 cycles in 0.1 M NaOH electrolyte (Figures S25–S27). Similar to the Co EXAFS analysis, the subtle changes of the coordination environment of the Ni-intercalated ions (Figure S28) suggest that they retain their local environment, without undergoing irreversible oxidation or leaving the interlayers.

**Figure 4:**
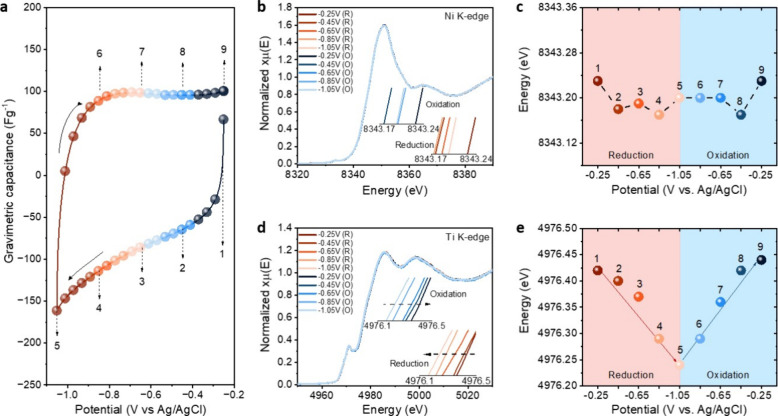
(a) CV of Ni_0.31_Ti_3_C_2_T_*x*_ at 1 mVs^–1^. Electrochemical XAS data of Ni_0.31_Ti_3_C_2_T_*x*_ where (b) Ni K-edge XANES spectra collected during negative and positive potential scans and (c) changes of Ni edge energy with applied potentials. (d) Ti K-edge XANES spectra during charging and discharging and (e) variation of Ti edge energy at different applied potentials.

This observation underscores an intriguing phenomenon: transition metals (TMs) typically considered redox-active may not exhibit the expected redox behavior when interacting with an MXene host. These three scenarios presented in this and our previous work reveal key implications: (1) due to their unique properties, as well as the distinct and confined local coordination environment between the MXene interlayers—defined by the positions of TM ions, coordination numbers, local symmetry, and surrounding species—result in varying interactions with the MXene matrix, thereby influencing their individual contributions to charge storage. (2) Regardless of the intercalated TM, the redox of the Ti-based MXene host consistently dominates the overall capacitance.

Additionally, the DOS calculated above without an applied potential cannot be directly correlated to the electrochemical behaviors of TM-MXenes as it represents a static condition and does not account for the dynamic changes occurring during electrochemical cycling. Under applied potentials, the Fermi levels of the TM-Mxene electrodes could potentially shift, bringing electronic states that were initially far from the Fermi level to more electrochemically accessible for redox reactions.

## Conclusions

Our findings demonstrate that the choice of intercalated metal significantly influences MXene properties, notably electrical conductivity and charge storage behavior. We observed that Ti atoms within the MXene layers become oxidized to varying degrees depending on the intercalated transition metal, altering the electronic density of states near the Fermi level. Unlike our previous study, where Cu ions underwent partial reduction upon intercalation, the intercalated Ni, Co, Mn, and Zn ions investigated here consistently maintained their +2 oxidation states. AIMD and DFT further revealed distinct spatial arrangements and coordination environments of these intercalated ions, significantly impacting their interactions with MXene surfaces and influencing their electronic structure.

Moreover, in situ XAS measurements revealed distinct charge storage mechanisms: Co ions actively participate in pseudocapacitive processes alongside Ti atoms, whereas Ni ions remain electrochemically inactive, leaving Ti atoms as the sole redox-active species. These insights highlight the complex interplay between MXenes and transition metal intercalants, offering a potential to tune MXene electronic and electrochemical properties through careful selection of transition metal intercalants.

## Materials and Methods

### Synthesis of Ti_3_C_2_T_*x*_

Both TM-intercalated Ti_3_C_2_T_*x*_ and Ti_3_C_2_T_*x*_ MXenes were produced following established steps.^17,22,23^ To begin, 3 g of Ti_3_AlC_2_ powder (Carbon-Ukraine, particle size <44 μm) was slowly introduced into a 30 mL etchant solution containing 10 wt % hydrofluoric acid (HF, Sigma-Aldrich, 48 wt %) and approximately 3.3 g of LiCl, maintaining a molar ratio of 5:1 relative to Ti_3_AlC_2_. During this process, the Al layers were etched, and Li^+^ ions were intercalated into the MXene layers. The solution was then mixed continuously at 350 rpm for 24 h at 25 °C. Following this, the resulting wet sediment was roughly split into six portions, each containing 0.5 g of MXene. Each portion underwent three rounds of washing with 40 mL of 6 M hydrochloric acid (HCl, Sigma-Aldrich, 37%), with centrifugation at 3500 rpm for 5 min, discarding the supernatant after each wash. Then, the wet sediment was subjected to multiple washes with Mili-Q water until a pH of at least 5 was achieved. The nonintercalated Ti_3_C_2_T_*x*_ powder, referred to as pristine MXene, was collected after these washes, dried via vacuum-assisted filtration, and used for subsequent characterization.

### Preparation of TM-Intercalated Ti_3_C_2_T_*x*_

Transition metal cation intercalation into Ti_3_C_2_T_*x*_ was carried out by mixing 0.5 g of wet Ti_3_C_2_T_*x*_ sediment with 40 mL of 0.1 M, 0.5 M, 1 M nickel­(II) chloride hexahydrate (NiCl_2_·6H2O, Sigma-Aldrich, ≥98%); 0.5 M, 1 M cobalt­(II) chloride hexahydrate (CoCl_2_·6H_2_O, Sigma-Aldrich, 98%); 0.1 M zinc­(II) chloride (ZnCl_2_, Sigma-Aldrich, ≥98%); and 0.1 M manganese­(II) chloride tetrahydrate (MnCl_2_·4H_2_O, Sigma-Aldrich, ≥98%). After shaking the mixtures for 5 min and settling for 1 h, the supernatant was discarded via centrifugation at 3500 rpm for 5 min. Fresh intercalation solutions were introduced, and the mixtures were stirred at 300 rpm under an Ar atmosphere for 24 h at room temperature. The intercalated Ti_3_C_2_T_*x*_ wet sediments were rinsed thoroughly with Mili-Q water three times and vacuum-dried for 24 h.

### Electrode Preparation

The preparation of the working electrode (WE) and counter electrode (CE) followed methods outlined in references.^4,24^ The WE was made of 90 wt % of MXene powder, 5 wt % of polytetrafluoroethylene binder (PTFE, Sigma-Aldrich), and 5 wt % of carbon black (CB, Orion). These components were mixed with excess ethanol using an agate mortar and pestle to form a uniform slurry. Once the ethanol evaporated, the resulting dried slurry was transferred to a clean glass surface. By adding a few drops of ethanol, the material was pressed and rolled mechanically into a free-standing film. The CE, comprising 95 wt % of activated carbon (MTI) and 5 wt % of polytetrafluoroethylene binder (PTFE, Sigma-Aldrich), was prepared using a similar procedure.

X-ray diffraction measurements were conducted using Cu K_α_ radiation (λ = 1.5418 Å, 40 mA and 40 kV) on a PANalytical Empyrean X-ray Powder Diffractometer. Si powder was mixed with all samples, except the pristine Ti_3_C_2_T_*x*_. The powders were scanned in reflection mode over a 2θ range from 4.5° to 60°, with a duration of 500 s per each 0.067° acquisition step.

Scanning transmission electron microscopy (STEM) imaging coupled with energy-dispersive X-ray spectroscopy (EDX) mapping was acquired using an FEI Talos F200X microscope equipped with an FEI SuperX detector (Chem S/TEM, ScopeM, ETH Zurich), at 200 kV for 5 min. Prior to acquisition, the powders were ground and dusted onto nickel or copper mesh lacey carbon support films (EM resolutions, Quantifoil).

Scanning electron microscopy (SEM) images and EDX analyses were acquired at 20 kV for 5 min, utilizing a Hitachi S-4800 microscope. The MXene powder was spread onto the carbon tape, with excess powder removed using compressed air.

Inductively coupled plasma–optical emission spectrometry (ICP–OES) measurements were carried out using an Agilent 720 ES instrument. Approximately 5–8 mg of TM-intercalated-Ti_3_C_2_T_*x*_ powder was dissolved in 5 mL of 20 wt % HNO_3_ (Sigma-Aldrich, 70%) and stirred at 100 rpm for a minimum of 24 h. After complete dissolution of the MXene powder, the solution was then diluted to 10 wt % HNO_3_ for analysis. Instrument calibrations were conducted before each measurement using solutions of CoSO_4_ (Sigma-Aldrich, ≥99%), NiSO_4_ (Sigma-Aldrich, ≥98%), CuSO_4_ (Carl Roth, 99%), ZnSO_4_ (Sigma-Aldrich, 99%), and MnCl_2_ (Sigma-Aldrich, ≥97%) prepared in 10 wt % of HNO_3_ at TM ion concentrations of 0, 10, 50, and 100 ppm, respectively.

Resistivity measurements were performed with an Ossila four-point probe instrument. About 25 mg of MXene powder was pressed using a hydraulic pellet press at 15 MPa, yielding 6 mm-diameter and approximately 0.25 mm-thick pellets.

### Electrochemical Setup and Measurements

Cyclic voltammetry (CV) was carried out in a three-electrode Swagelok cell configuration using Biologic MPG-200. The WE (a free-standing MXene electrode) was placed on a glassy carbon current collector (CH instruments), while the CE (free-standing activated carbon) was placed on a Ti rod current collector. The reference electrode employed was Ag/AgCl in 1 M KCl (CH instruments), with 0.1 M NaOH (Sigma-Aldrich, ≥98%) as the electrolyte and a polypropylene membrane (Celgard 3501) as the separator.

Ex-situ X-ray absorption spectroscopy (XAS) measurements were carried out at the P64 Advanced X-ray Absorption Spectroscopy beamline located at Deutsches Elektronen (DESY) Synchrotron (Hamburg, Germany). The sample preparation steps were as follows: approximately 4 mg of powder sample was mixed homogeneously with about 70 mg of cellulose (Sigma-Aldrich) in an agate mortar. The well-mixed powder was pressed at a 5-ton-force by a pelletizer die set, forming a pellet with a thickness of 1 mm and a diameter of 13 mm. Using a Si (111) monochromator, the pellets were assessed in transmission mode for the Ti K-edge; in fluorescence mode with a passivated implanted planar silicon (PIPS) detector for the TM K-edges. Before measuring the pellets, energy calibration was carried out using standard metal foils corresponding to Co (edge at 7.70 keV), Ni (edge at 8.33 keV), Cu (edge at 8.97 keV), Mn (edge at 6.53 keV), Zn (edge at 9.65 keV), and Ti (edge at 4.96 keV). Subsequently, signals for each pellet were obtained through five acquisitions at the respective K-edges, with each acquisition lasting approximately 2 min.

In situ X-ray absorption spectroscopy (XAS) measurements for Co–Ti_3_C_2_T_*x*_ were carried out at the P64 Advanced X-ray Absorption Spectroscopy beamline at the DESY Synchrotron (Hamburg, Germany), while measurements for Ni–Ti_3_C_2_T_*x*_ were conducted at the B18 X-ray Absorption Spectroscopy beamline at the Diamond Light Source Synchrotron (Didcot, UK). Ti and Co K-edge signals for Co–Ti_3_C_2_T_*x*_ were collected in fluorescence mode employing a Si (111) monochromator and a PIPS detector. For the Ni–Ti_3_C_2_T_*x*_ sample, Ti signals were recorded using a 4-element Si drift fluorescence detector, while during the collection of Ni signals, a 36-element Ge fluorescence detector was used. During data acquisition, the detectors were positioned at a 45° angle relative to the MXene electrode within a three-electrode-configuration ECC-Opto-Std test cell (EL-cell, Germany). The in situ cell incorporated a 50-um-thick poly­(ether imide) sheet coated with 50 nm Au, which served as the current collector and X-ray window for the WE. Similar to previously described electrode preparation steps, the WE consisted of 90 wt % of Co–Ti_3_C_2_T_*x*_ or Ni–Ti_3_C_2_T_*x*_ powder, 5 wt % of PTFE, and 5 wt % of CB. The CE was overcapacitive activated carbon, the reference electrode was an eDAQ leakless Ag/AgCl (with a filling electrolyte 3.4 M KCl), and the separator was a Celgard 3501 polypropylene membrane. The in situ cell was cycled for three CV cycles at 1 mVs^–1^ within specific potential ranges of interest (−0.4 V to −1.0 V for Co–Ti_3_C_2_T_*x*_; and −0.25 V to −1.05 V for Ni–Ti_3_C_2_T_*x*_) before XAS data acquisition. Then, linear sweep voltammetry (LSV) and approximately 15 min of chronoamperomtery (CA) were executed for each relevant potential. A minimum of three spectra were acquired at each potential hold, with each acquisition lasting 3 min.

Next, Athena software was used to carry out XAS data analysis. The TM and Ti K-edge energies were determined as the energy at the half-height of the normalized intensity of the spectrum,^2,25,26^ aligning with the zero of the second derivative.^26–28^ The Ti oxidation states in Co/Ni–Ti_3_C_2_T_*x*_ samples were inferred by correlating Ti edge energy with the Ti valence of TiO (Alfa Aesar, 99.5%) and TiO_2_ rutile (Sigma-Aldrich, 99.5%) reference compounds. Similarly, the evaluation of Co or Ni oxidation states involved referencing CoO (Sigma-Aldrich, 99.99%) and Co foil or NiO (Sigma-Aldrich, ≥ 99.99%) and Ni foil.

Oxidation state changes observed for Co and Ti (in Co–Ti_3_C_2_T_*x*_), as well as Ti (in Ni–Ti_3_C_2_T_*x*_), were corroborated with capacitance values derived from CV measurements. The reaction involved can be expressed as

$${\mathrm{TM}}_{x}{\mathrm{Ti}}_{3}C_{2}O_{x}(\mathrm{OH}{)}_{y}F_{2-x-y}+\delta e^{-}+\delta {\mathrm{Na}}^{+}\to {\mathrm{TM}}_{x}{\mathrm{Ti}}_{3}C_{2}O_{x}(\mathrm{OH}{)}_{y}F_{2-x-y}{\mathrm{Na}}_{\delta }$$

The capacitance contributions from Ti and TM were calculated using the following formula: $C_{g}=\frac{zF}{M_{w}V}$ , where *C*_*g*_ is the gravimetric capacitance in F g^–1^, *z* is the number of electrons involved, F is the Faraday’s constant (96,485 C mol^–1^), *M*_w_ is the molar weight in g mol^–1^, and V refers to the potential window in V.

In the case of Co_0.20_Ti_3_C_2_T_*x*,_ the contributions from Co redox involved 0.22 × 0.2 = 0.044 electrons, and for Ti, the value was 0.026 × 3 = 0.78 electrons. The estimated capacitance of 90 F g^–1^ (with *M*_w_ = 213.8 g mol^–1^) closely matches the value of 93 F g^–1^ derived from CV data. This confirms that the charge storage mechanism of Co_0.20_Ti_3_C_2_T_*x*_ involves contributions from both Co and Ti redox processes. For Ni_0.31_Ti_3_C_2_T_*x*,_ the electrons participating in Ti redox were estimated to be 0.06 × 3 = 0.18. The calculated capacitance of 98 F g^–1^ (with *M*_w_ = 220.8 g mol^–1^) aligns well with the CV capacitance of 102 F g^–1^, indicating that the charge contribution correlates only to Ti redox.

### Density Functional Theory (DFT) Calculation and Molecular Dynamics (MD) Simulations

Although we have different cations and the calculation setup can vary slightly, all calculations generally involve the following three steps: (1) creating the structural model; (2) structural optimization; and (3) ab initio molecular dynamics (AIMD) simulation and properties analysis. In the following paragraphs, we provide a comprehensive explanation of the above steps.

### Initial Structure Generation

As detailed in our previous work,^17^ we first reduced the cell size for affordable computational cost. The reduced cell has 3 formula units of Ti_3_C_2_(OH)_2_ and H_2_O molecules that have been removed. The initial *c* lattice parameter is 12.83 Å. In our previous Cu-inserted MXene, we determined that the concentration of Cu^2+^ is 0.23 per formula unit, which corresponds to 2 Cu^2+^ ions inserted. Therefore, for all other cations, we fixed the same number of cations to 2. The initial water layer was built using Packmol.^29^ We treated the two cations as Na^+^ ions and solvated the Na^+^ ions in H_2_O. Then, we equilibrated the box using a classical force field in LAMMPS.^30^ Subsequently, the equilibrated water layer was inserted into the Mxene layer, and the Na^+^ ions were substituted with the corresponding cations. Four protons were randomly removed to implicitly assign a +2 charge to the cations. Then, we performed structural relaxation on the models.

### Structural Optimization

All our electronic structure calculations were performed within the density functional theory framework, as implemented in the Vienna ab initio simulation package (VASP).^31,32^ The wave functions were expanded in plane wave basis sets, and the projector augmented wave (PAW) method was used to describe the core–valence interactions. The exchange-correlation energy was approximated with the generalized gradient approximation as formulated by Perdew, Burke, and Ernzerhof (PBE).^33,34^ For all of the structural optimizations, we set the kinetic energy cutoff to 520 eV and sampled the Brillouin zone with a *k*-spacing of 0.3 Å^–1^. The electronic convergence threshold was set to 10^–6^ eV, and the forces were converged to 0.05 eV Å^–1^. The number of water molecules was then fixed by matching the lattice parameter with experimental values. For Cu^2+^-inserted MXene, we found that 10 H_2_O molecules yield the best agreement with experimental data, with a *c* value of 14.8 Å, while for Ni^2+^, Co^2+^, and Mg^2+^, the resulting structures all have 11 H_2_O molecules, with *c* lattice parameters being 14.87, 14.78, and 14.84 Å respectively. It should be noted that the effect of dispersion was tested on hydrated MXene by including the D3 correction.^35^ The difference in the *c* lattice parameter was smaller than 3%, so that it was decided not to include the correction in further calculations.

### Ab Initio Molecular Dynamics

The AIMD calculations were performed with the NVT ensemble, where we fixed the number of atoms, volume, and temperature to 300 K, with a time step of 1 fs. The gamma point was used to sample the Brillouin zone, with a reduced cutoff energy of 450 eV to lower the computational cost. The calculations involved three steps: (1) equilibration for 2 ps; (2) first production run for 40 ps; and (3) second production run for 30 ps. Inspecting the final structure from the second step, we found the existence of isolated protons and H_2_ gas molecules. We speculated that they originate from an excess of H atoms in our setup, which should not exist. Therefore, in the last step, we manually removed them before the last production run. The final structures from AIMD were used to calculate the density of states (DOS) and Bader charges.^36^

## Supplementary Material



## Notes

Radial distribution function and coordination number of intercalated TM cations; Ni_0.31_Ti_3_C_2_T_*x*_ AIMD simulation with electronic charge distribution; density of states of pristine and TM-intercalated Ti_3_C_2_T_*x*_ calculated using PBE; XRD data for pristine and TM-intercalated Ti_3_C_2_T_*x*_; TM ions loading obtained from ICP–OES and SEM–EDX; STEM–EDX of TM-Ti_3_C_2_T_*x*_; Ti K-edge XANES of pristine and TM-intercalated Ti_3_C_2_T_*x*_; CV data for Co_0.20_Ti_3_C_2_T_*x*_, Ni_0.31_Ti_3_C_2_T_*x*_ and Ti_3_C_2_T_*x*_ MXenes; Co and Ni K-edge XANES of Co_0.20_Ti_3_C_2_T_*x*_ and Ni_0.31_Ti_3_C_2_T_*x*_, respectively; estimation of Co and Ti oxidation state through XAS data; Co and Ni loading, as well as XRD of Co_0.20_Ti_3_C_2_T_*x*_ and Ni_0.31_Ti_3_C_2_T_*x*_ before and after electrochemical cycling; EXAFS analysis of Co and Ni; first solvation shell distance and coordination number of TM ions within MXenes and in bulk solution; final composition of different simulated MXene systems; and Bader charge analysis data for all measured MXenes (PDF)
