# Supplementary material for: Intercalation of Transition Metals into MXenes: Impact on Electronic and Pseudocapacitive Properties
Source: ACS Nano. 2025 Sep 5;19(36):32183–91. doi: 10.1021/acsnano.5c06170 (PMC12444977; doi:10.1021/acsnano.5c06170)
Supplement: Supplementary file 1 [file nn5c06170_si_001.pdf]

Intercalation of Transition Metals into MXenes: Impact on Electronic and Pseudocapacitive Properties

Shianlin Wee[1], Xiliang Lian[2,3], Dario Gomez Vazquez[1], Mathieu Salanne[2,3,4*], Maria R. Lukatskaya[1*]

1. Electrochemical Energy Systems Laboratory, Department of Mechanical and Process Engineering, ETH Zurich, 8092 Zurich, Switzerland.
2. Sorbonne Université, CNRS, Physicochimie des Électrolytes et Nanosystèmes Interfaciaux, F-75005 Paris, France.
3. Réseau sur le Stockage Electrochimique de l'Energie (RS2E), FR CNRS 3459, 80039 Amiens Cedex, France.
4. Institut Universitaire de France (IUF), 75231 Paris, France.

*Corresponding authors email: mathieu.salanne@sorbonne-universite.fr and mlukatskaya@ethz.ch

TABLE OF CONTENT

Figure S1 *a. Radial distribution function (RDF) and b. running coordination numbers (with all the oxygen atoms) of the metal ions. The plots corresponding to the Co, Ni, Cu, and Mg inserted MXenes are shown in wine, blue, yellow, green respectively.* 3

Figure S2 *Snapshot extracted from the Ni-MXene AIMD simulation. Panel (a) shows the position of the Ni atoms and panel (b) the electron depletion (cyan isosurface) and accumulation (yellow isosurface), respectively. The isosurface value is set to 0.008. The charge density difference is obtained as the following* ρNi-MXene-ρMXene-ρNi*, where ρ is the electronic charge density. It measures the electronic charge transfer upon Ni intercalation into the water layer.* 4

Figure S3 The *density of states for a. pristine MXenes and b. Mg, c. Co, d. Ni, and e. Cu-inserted MXenes calculated using PBE.* 5

Figure S4. *XRD patterns of pristine $Ti_3C_2T_x$ and $Ti_3C_2T_x$ intercalated with Co, Ni, Cu, Mn, Zn transition metal cations at different loading levels. Crystalline Si was added as internal standart.* 6

Figure S5. *The loading of intercalated Co, Ni, Mn, Zn ions were quantified through ICP-OES and SEM-EDX.* 7

Figure S6. *STEM-EDX of $Co_{0.20}Ti_3C_2T_x$ multilayer particles.* 8

Figure S7. *STEM-EDX of $Co_{0.09}Ti_3C_2T_x$ multilayer particles.* 9

Figure S8. *STEM-EDX of $Ni_{0.31}Ti_3C_2T_x$ multilayer particles.* 10

Figure S9. *STEM-EDX of $Ni_{0.13}Ti_3C_2T_x$ multilayer particles.* 11

Figure S10. *STEM-EDX of $Ni_{0.09}Ti_3C_2T_x$ multilayer particles.* 12

Figure S11. *STEM-EDX of $Mn_{0.09}Ti_3C_2T_x$ multilayer particles.* 13

Figure S12. *STEM-EDX of $Zn_{0.09}Ti_3C_2T_x$ multilayer particles.* 14

Figure S13. *The comparison of the Ti K-edges of all TMs-intercalated $Ti_3C_2T_x$.* 15

Figure S14. *The comparison of the cyclic voltammetry between $Co_{0.20}Ti_3C_2T_x$ and $Ti_3C_2T_x$ MXenes at 1 mVs^{-1} in 0.1M NaOH, respectively.* 16

Figure S15. *The XAS of $Co_{0.20}Ti_3C_2T_x$. The inset is the enlarged pre-edge region.* 17

Figure S16. *Average Co oxidation states at various potentials; Co K-edge energies of Co foil (0) and CoO are added for reference.* 18

Figure S17. *Average Ti oxidation states at various potentials; Ti K-edge energies of TiO (+2) and TiO_2 (+4) are added for reference.* 19

Figure S18. *The cyclic voltammetry of $Co_{0.20}Ti_3C_2T_x$ for 10,000 cycles at 10 mVs^{-1}.* 20

Figure S19. *The loading of Co in $Co_{0.20}Ti_3C_2T_x$ before and after 24-hour cycling, as well as after 10,000-cycle cycling at 10 mVs^{-1}, quantifying through SEM-EDX.* 21

Figure S20. *XRD diffraction patterns of $Co_{0.20}Ti_3C_2T_x$ before and after 24-hour cycling, as well as after 10,000-cycle cycling at 10 mVs^{-1}.* 22

Figure S21 *The EXAFS analysis of the Co ions of Co-$Ti_3C_2T_x$ without applied potential as well as at -0.4V and -1.0V respectively.* 23

Figure S22. *The comparison of the cyclic voltammetry between $Ni_{0.31}Ti_3C_2T_x$ and $Ti_3C_2T_x$ MXenes at 1 mVs^{-1} in 0.1M NaOH, respectively.* 24

Figure S23 *The XAS of $Ni_{0.31}Ti_3C_2T_x$. The inset is the enlarged pre-edge region.* 25

Figure S24. *Average Ti oxidation states at various potentials; Ti K-edge energies of TiO (+2) and TiO_2 (+4) are added for reference.* 26

Figure S25. *The cyclic voltammetry of $Ni_{0.31}Ti_3C_2T_x$ for 10,000 cycles at 10mVs^{-1}.* 27

Figure S26. *The loading of Ni in $Ni_{0.31}Ti_3C_2T_x$ before and after 24-hour cycling, as well as after 10,000-cycle of cycling at 10 mVs^{-1}, quantifying through SEM-EDX.* 28

Figure S27. *XRD diffraction patterns of $Ni_{0.31}Ti_3C_2T_x$ before and after 24-hour cycling, as well as after 10,000-cycle of cycling at 10 mVs^{-1}.* 29

Figure S28 *The EXAFS analysis of the Ni ions Ni-$Ti_3C_2T_x$ without applied potential as well as at -0.4V and -1.0V respectively.* 30

Table S1. *Final composition of the simulated systems* 31

Table S2. *Calculated first solvation shell distances and coordination numbers and experimental data in bulk solution.* 32

Table S3. *Bader charges of all elements.* 33

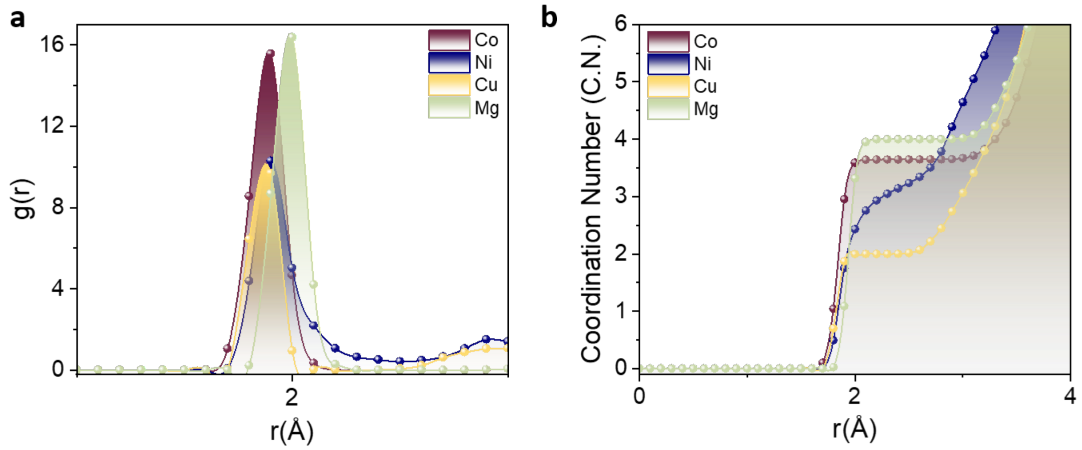

Figure S1 ***a.*** *Radial distribution function (RDF) and* ***b.*** *running coordination numbers (with all the oxygen atoms) of the metal ions. The plots corresponding to the Co, Ni, Cu, and Mg inserted MXenes are shown in wine, blue, yellow, green respectively.*

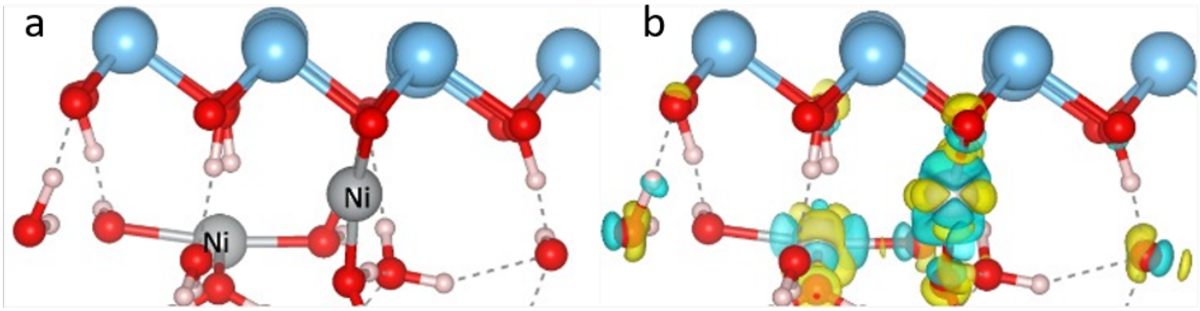

Figure S2 *Snapshot extracted from the Ni-MXene AIMD simulation. Panel (a) shows the position of the Ni atoms and panel (b) the electron depletion (cyan isosurface) and accumulation (yellow isosurface), respectively. The isosurface value is set to 0.008. The charge density difference is obtained as the following* $\boldsymbol{\rho}_{Ni-MXene} - \boldsymbol{\rho}_{MXene} - \boldsymbol{\rho}_{Ni}$, *where* $\boldsymbol{\rho}$ *is the electronic charge density. It measures the electronic charge transfer upon Ni intercalation into the water layer.*

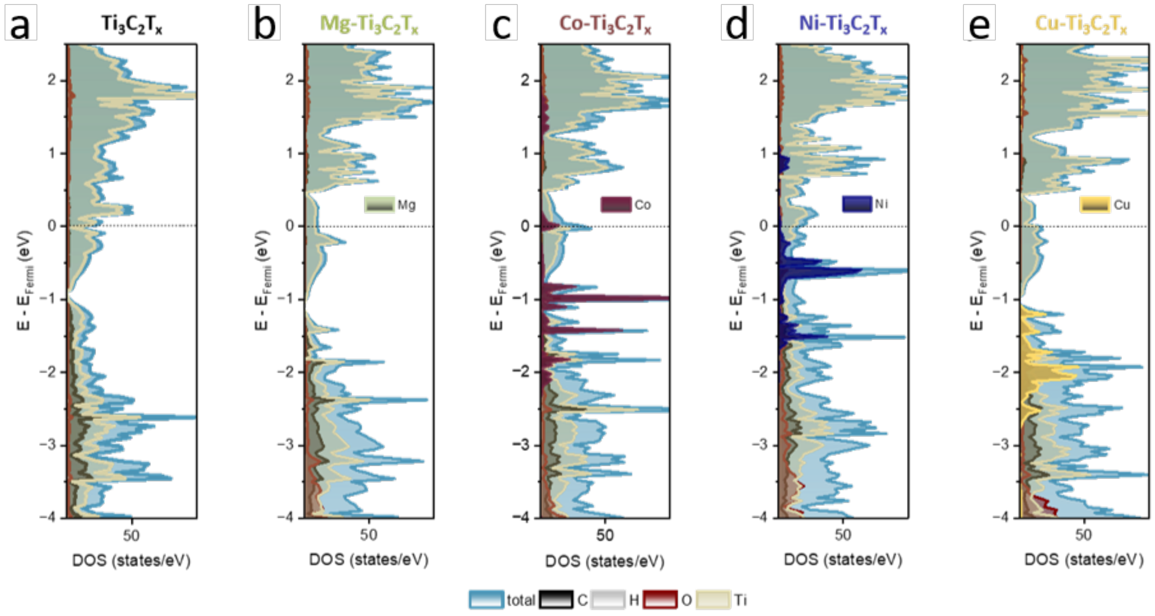

Figure S3 The *density of states for a. pristine MXenes and b. Mg, c. Co, d. Ni, and e. Cu-inserted MXenes calculated using PBE.*

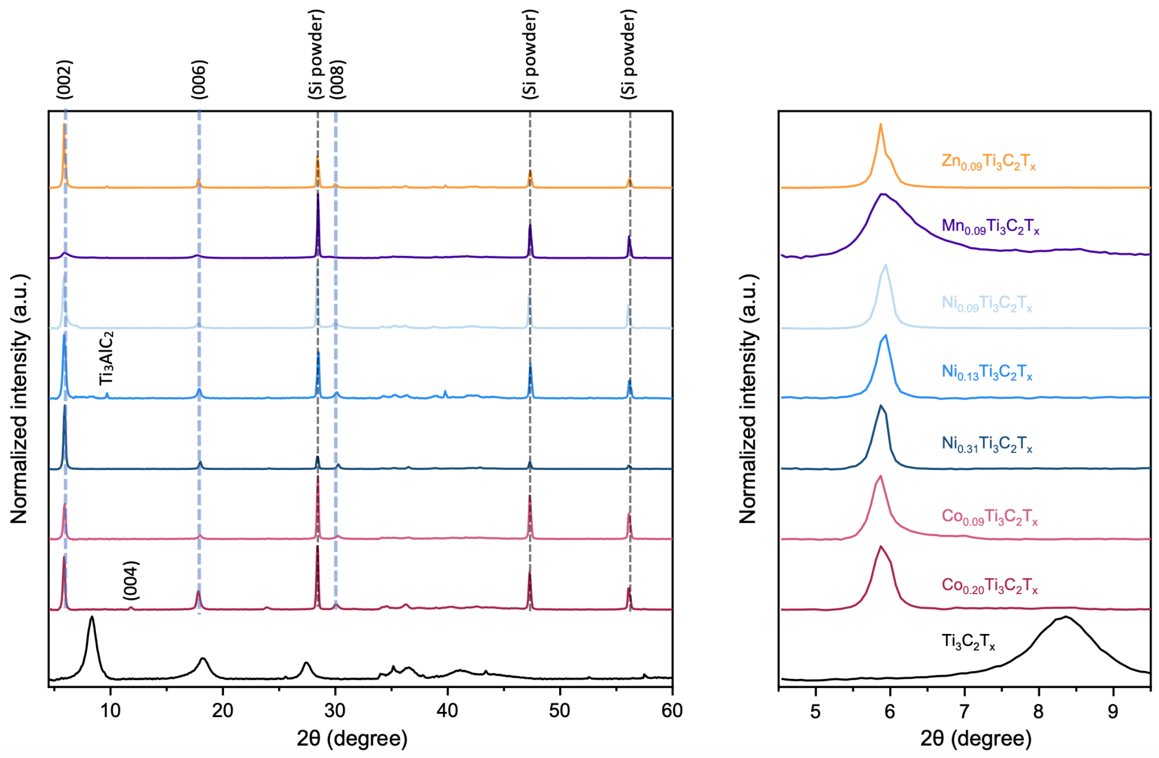

Figure S4. *XRD patterns of pristine $Ti_3C_2T_x$ and $Ti_3C_2T_x$ intercalated with Co, Ni, Cu, Mn, Zn transition metal cations at different loading levels. Crystalline Si was added as internal standart.*

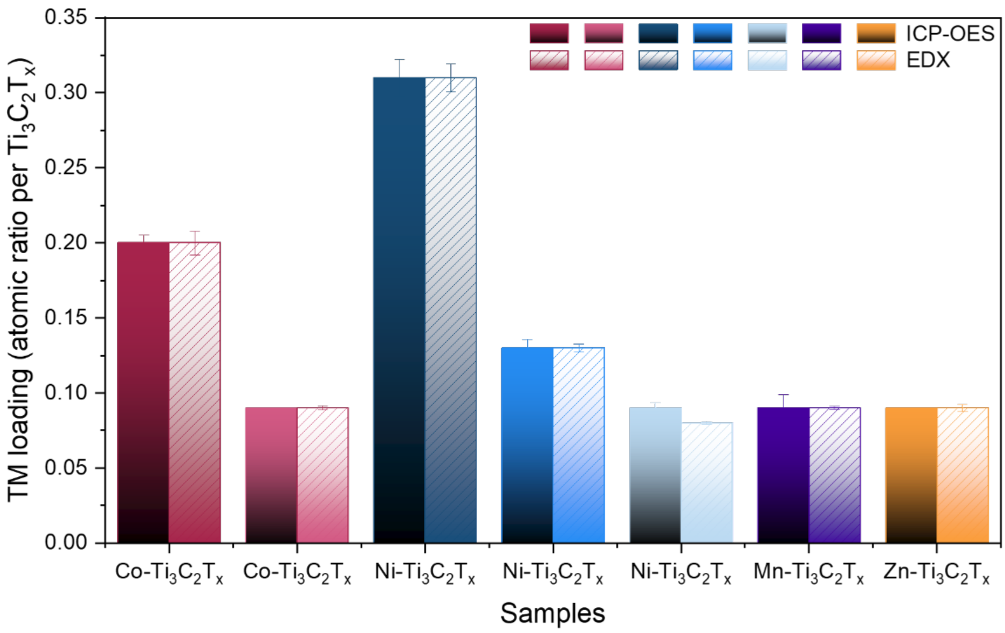

Figure S5. *The loading of intercalated Co, Ni, Mn, Zn ions were quantified through ICP-OES and SEM-EDX.*

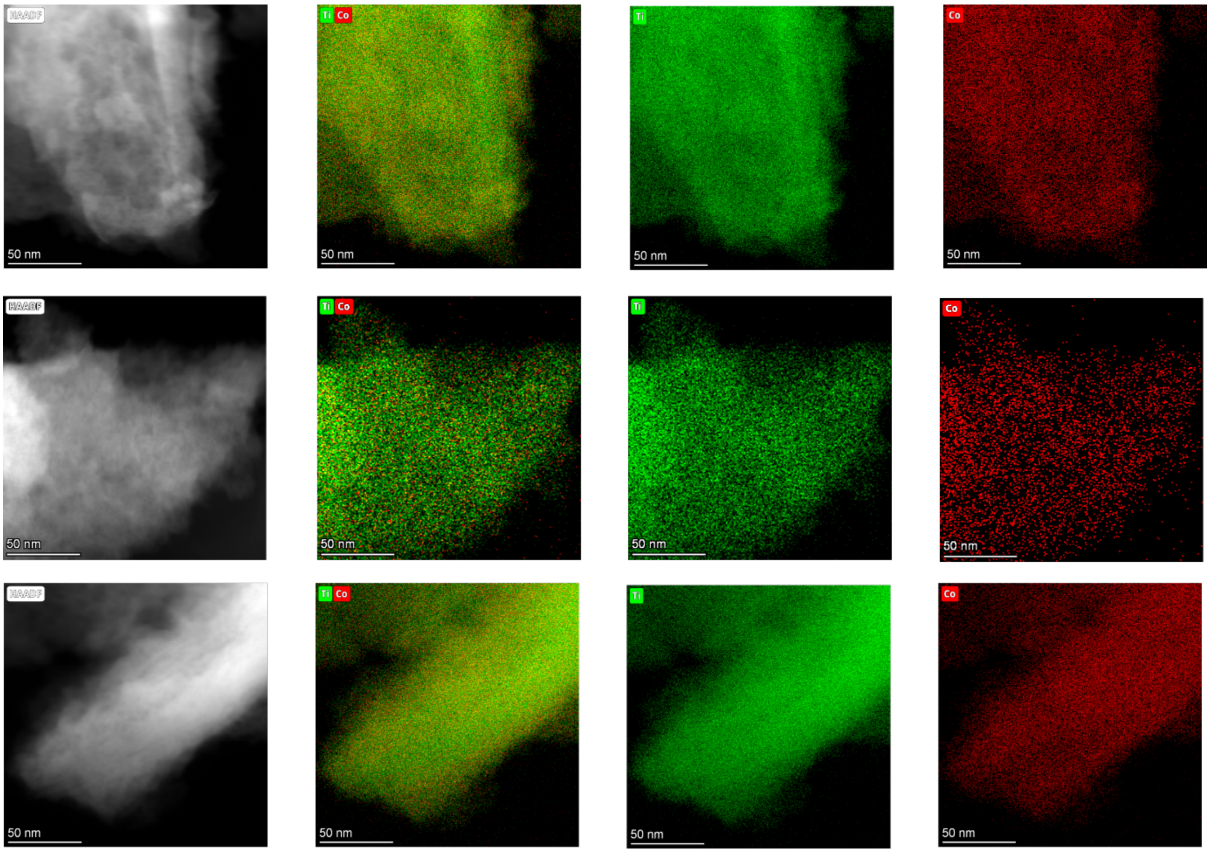

Figure S6. *STEM-EDX of $Co_{0.20}Ti_3C_2T_x$ multilayer particles.*

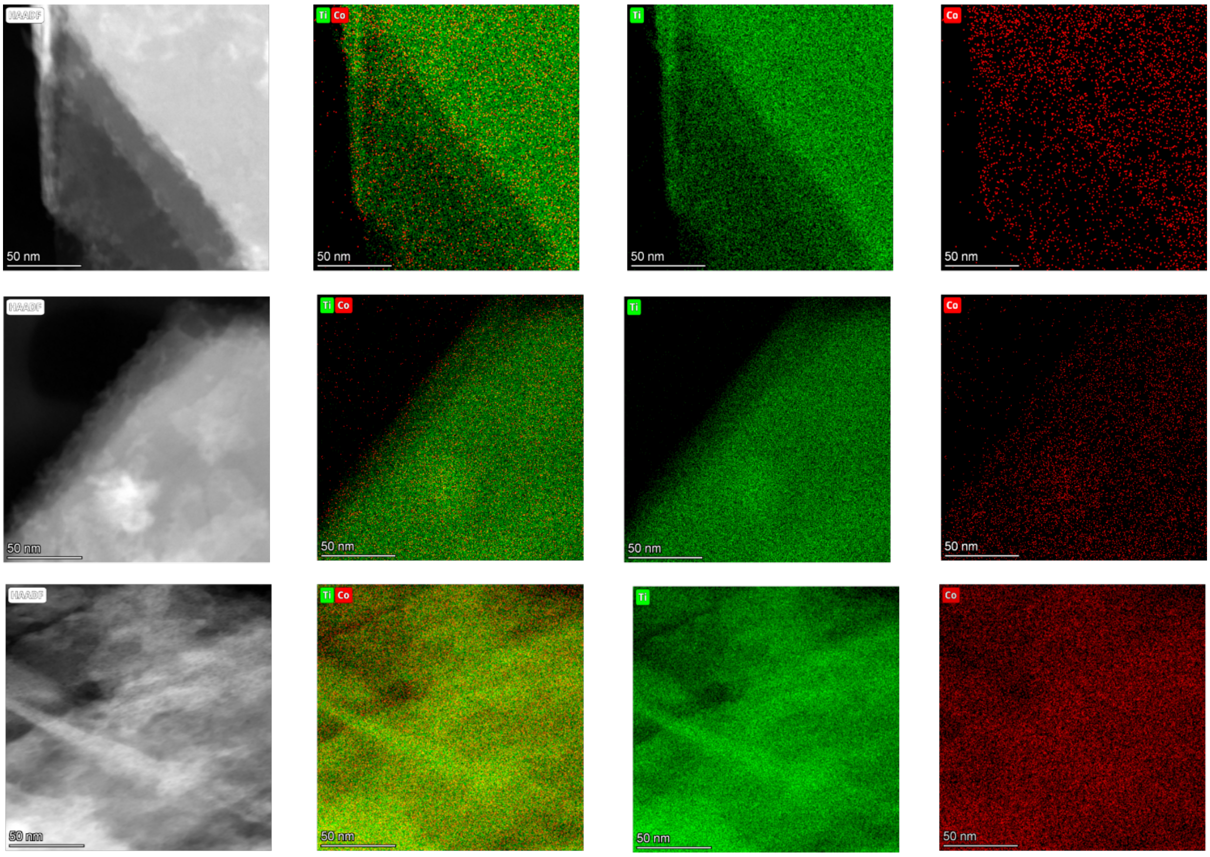

Figure S7. *STEM-EDX of $Co_{0.09}Ti_3C_2T_x$ multilayer particles.*

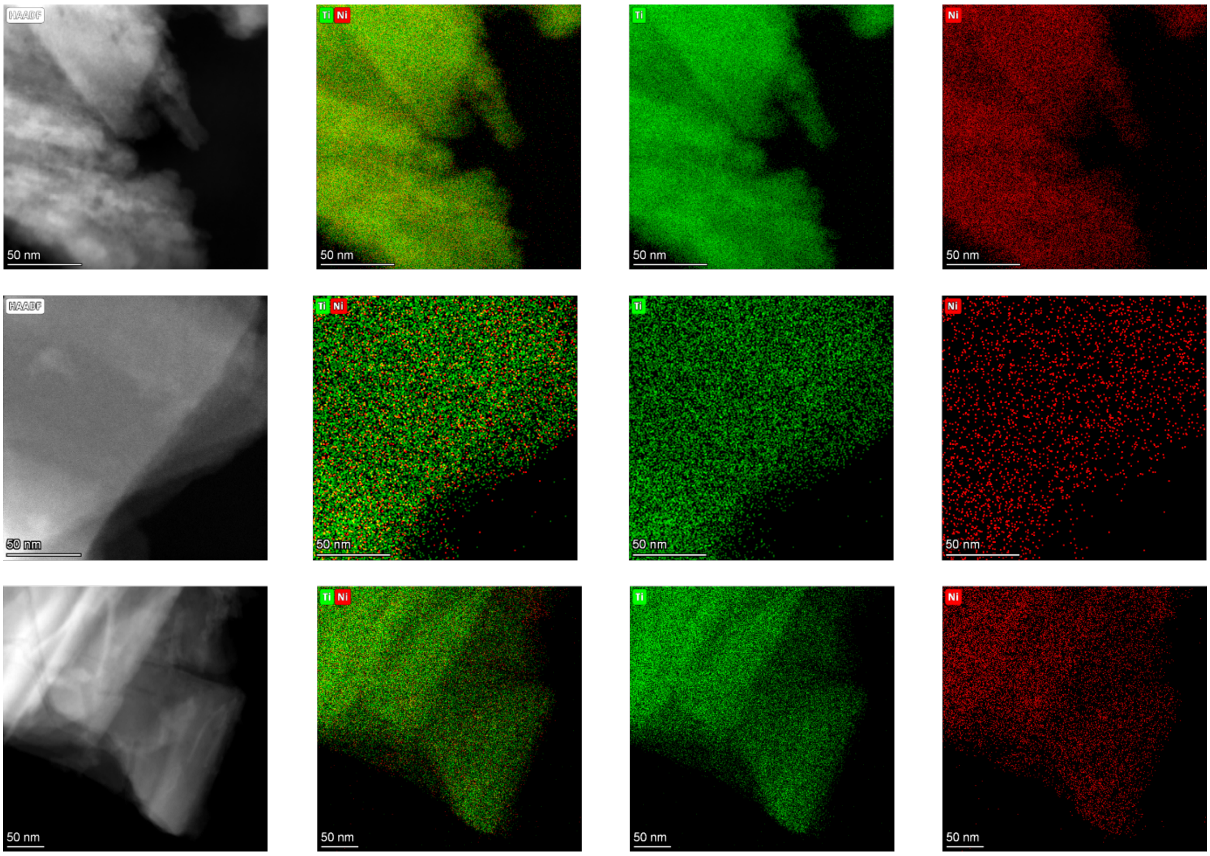

Figure S8. *STEM-EDX of $Ni_{0.31}Ti_3C_2T_x$ multilayer particles.*

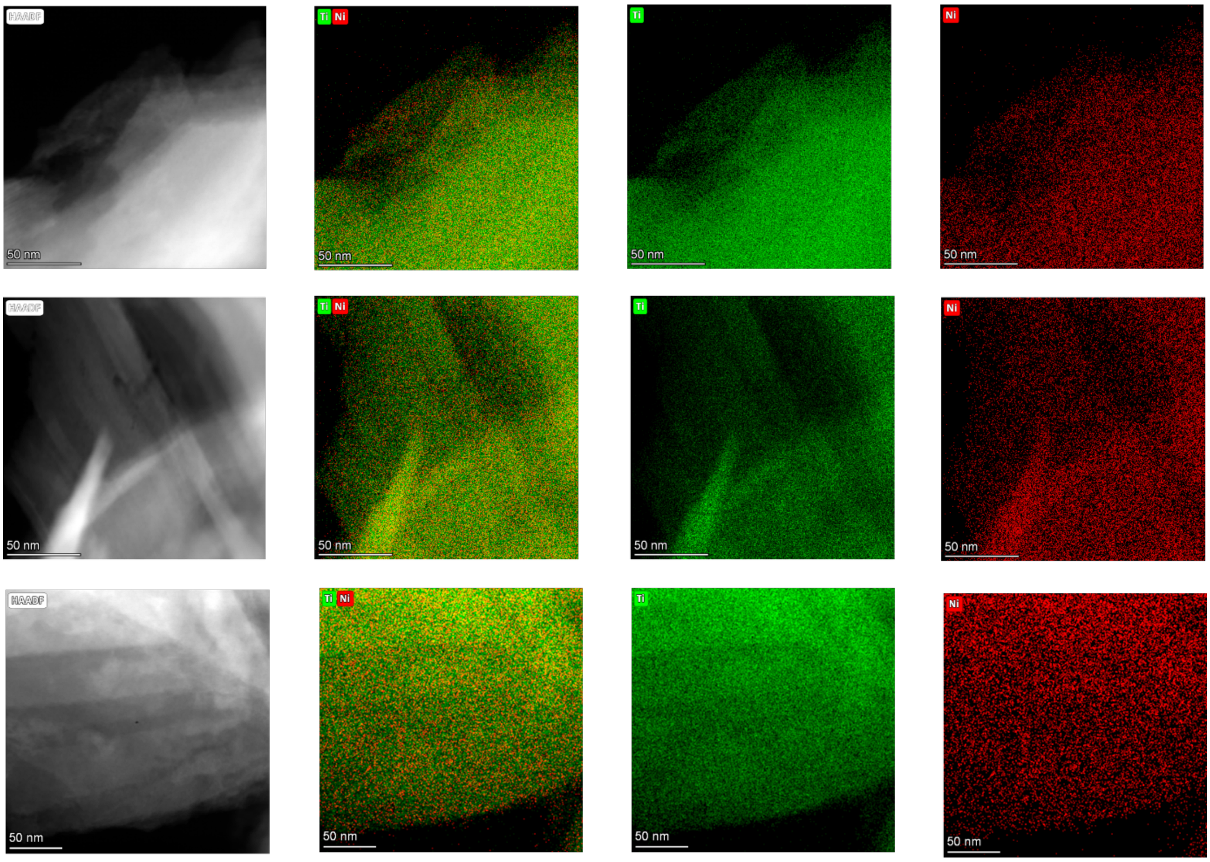

Figure S9. *STEM-EDX of $Ni_{0.13}Ti_3C_2T_x$ multilayer particles.*

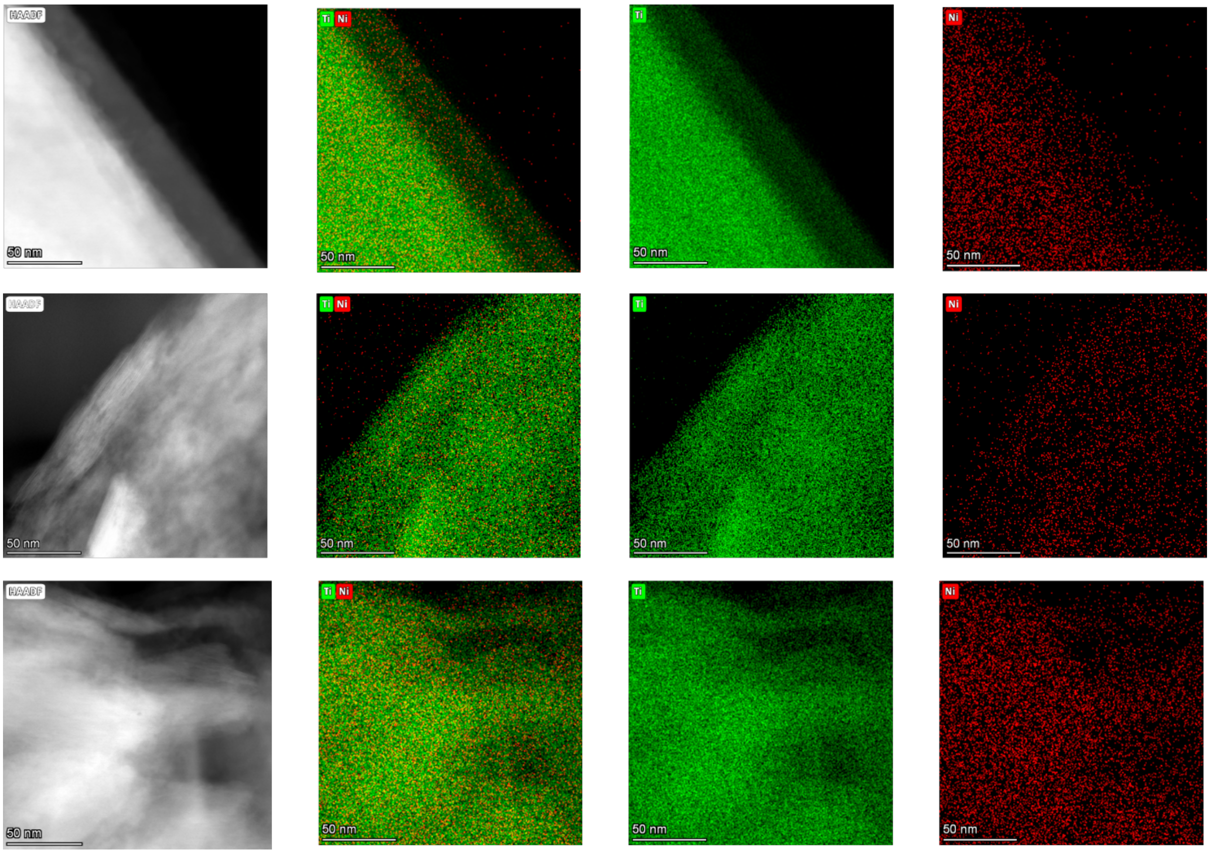

Figure S10. *STEM-EDX of $Ni_{0.09}Ti_3C_2T_x$ multilayer particles.*

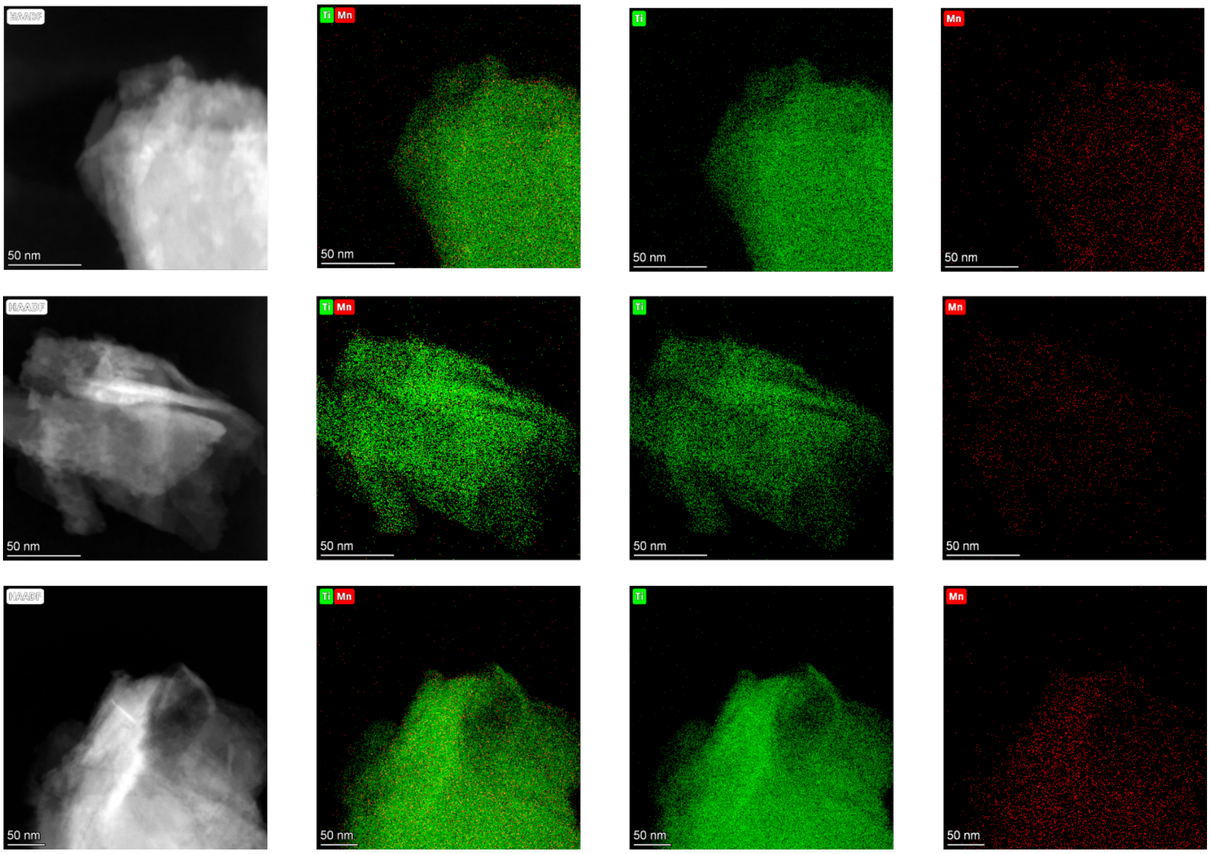

Figure S11. *STEM-EDX of $Mn_{0.09}Ti_3C_2T_x$ multilayer particles.*

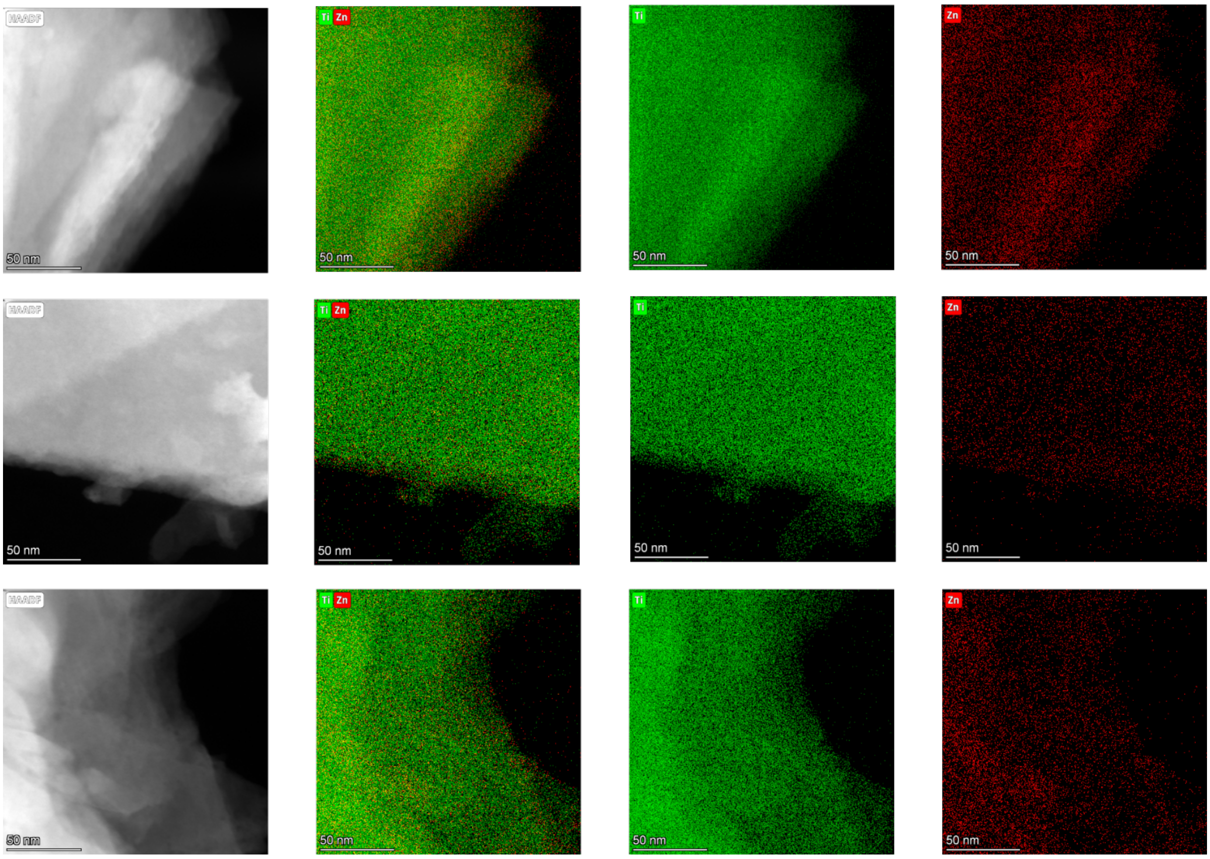

Figure S12. *STEM-EDX of* $Zn_{0.09}Ti_3C_2T_x$ *multilayer particles.*

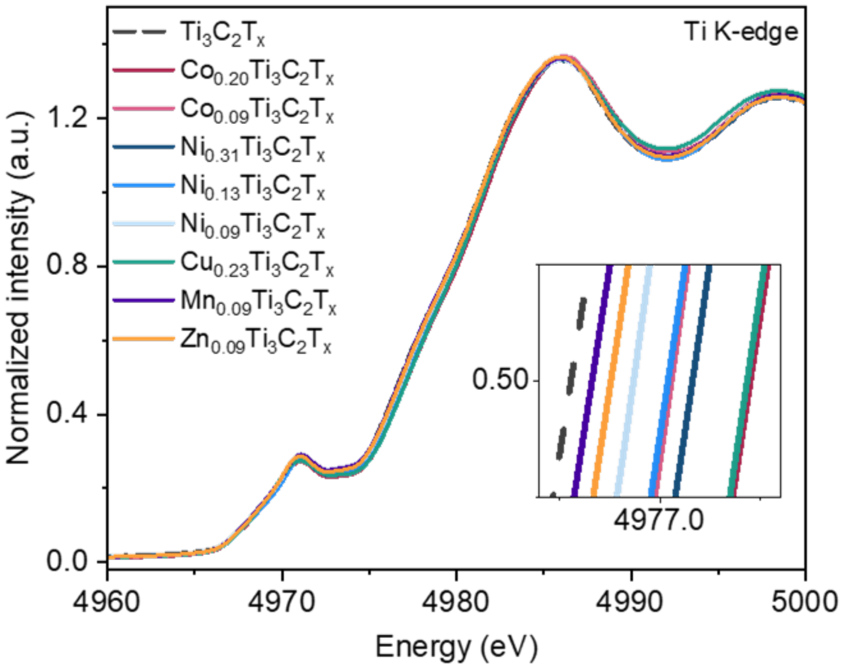

Figure S13. *The comparison of the Ti K-edges of all TMs-intercalated $Ti_3C_2T_x$.*

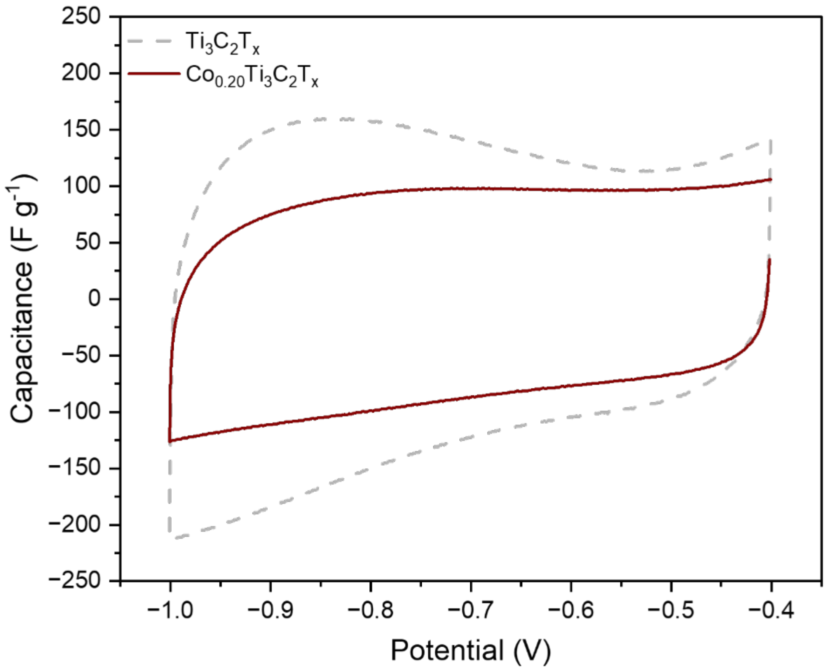

Figure S14. *The comparison of the cyclic voltammetry between $Co_{0.20}Ti_3C_2T_x$ and $Ti_3C_2T_x$ MXenes at 1 mVs^{-1} in 0.1M NaOH, respectively.*

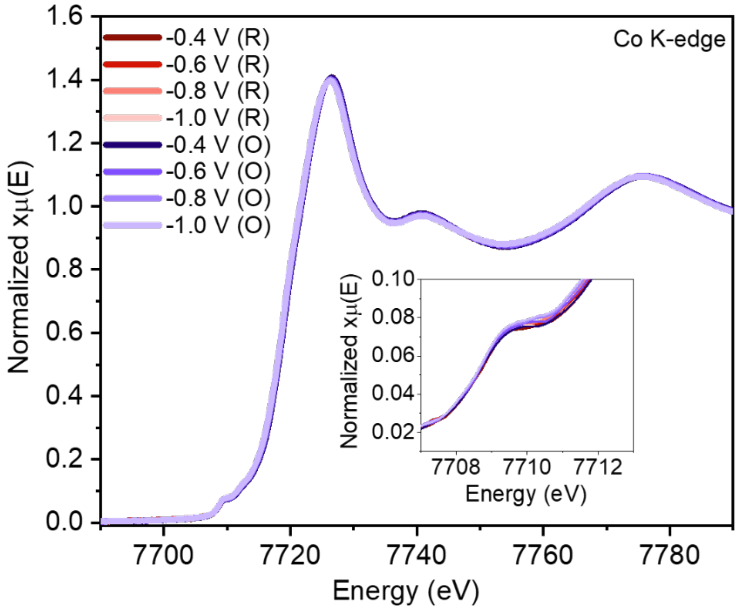

Figure S15. *The XAS of $Co_{0.20}Ti_3C_2T_x$. The inset is the enlarged pre-edge region.*

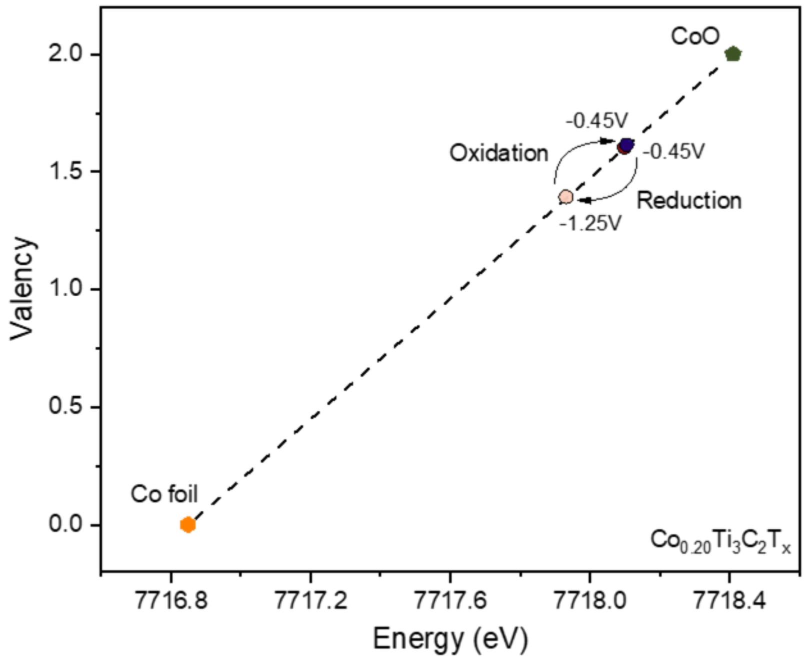

Figure S16. *Average Co oxidation states at various potentials; Co K-edge energies of Co foil (0) and CoO are added for reference.*

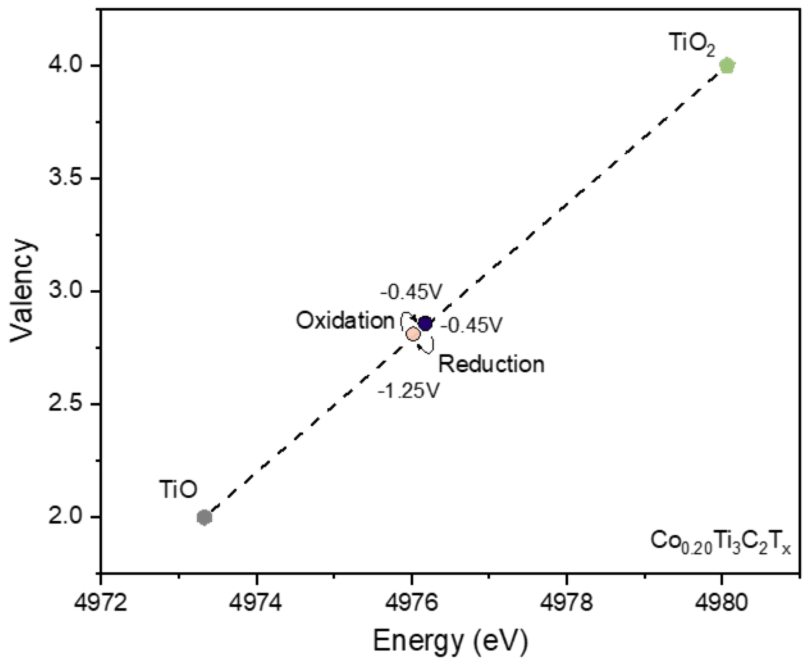

Figure S17. *Average Ti oxidation states at various potentials; Ti K-edge energies of TiO (+2) and TiO_2 (+4) are added for reference.*

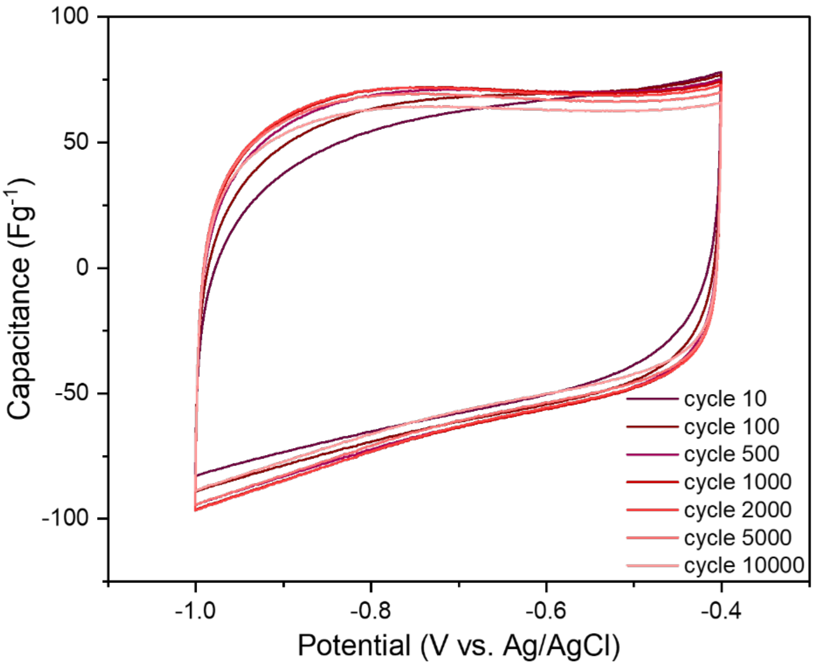

Figure S18. *The cyclic voltammetry of* $Co_{0.20}Ti_3C_2T_x$ *for 10,000 cycles at 10* mVs^{-1}.

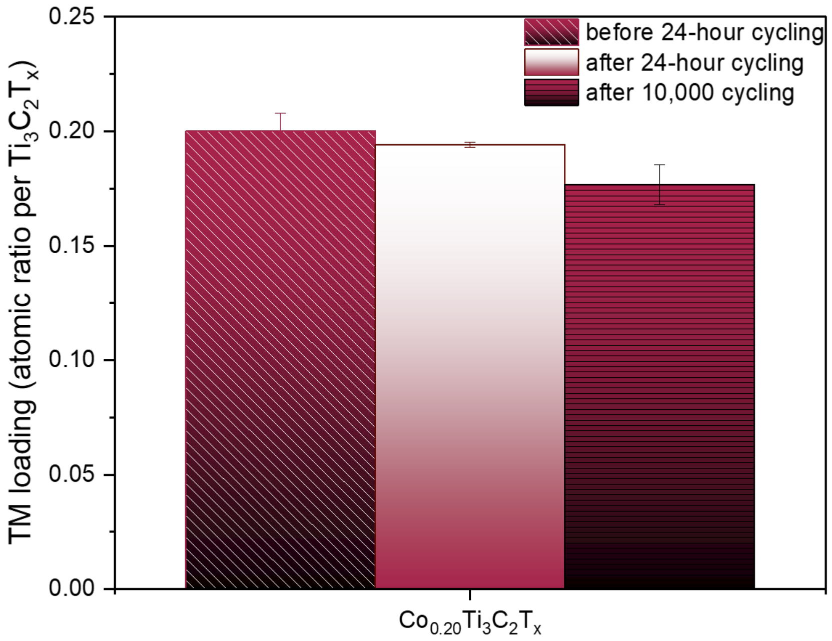

Figure S19. *The loading of Co in $Co_{0.20}Ti_3C_2T_x$ before and after 24-hour cycling, as well as after 10,000-cycle cycling at 10 mVs^{-1}, quantifying through SEM-EDX.*

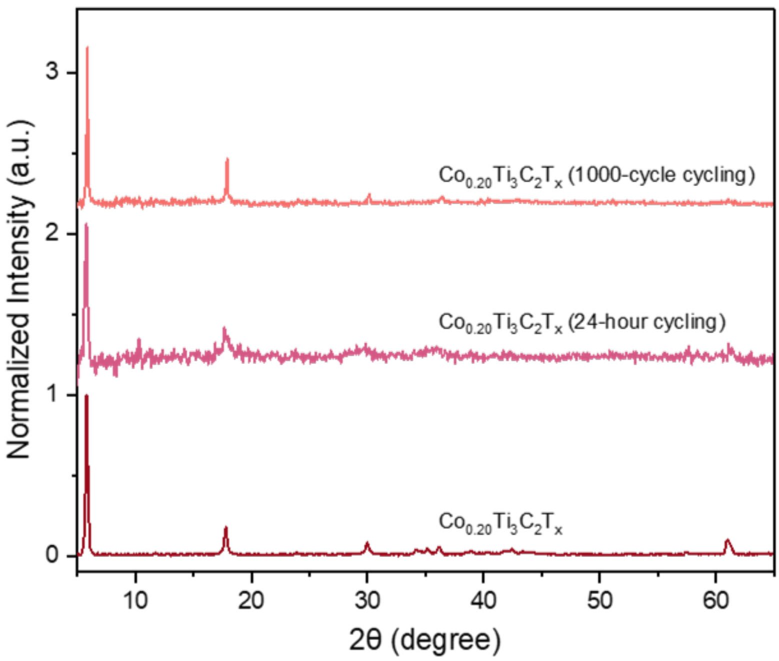

Figure S20. *XRD diffraction patterns of $Co_{0.20}Ti_3C_2T_x$ before and after 24-hour cycling, as well as after 10,000-cycle cycling at 10 mVs^{-1}.*

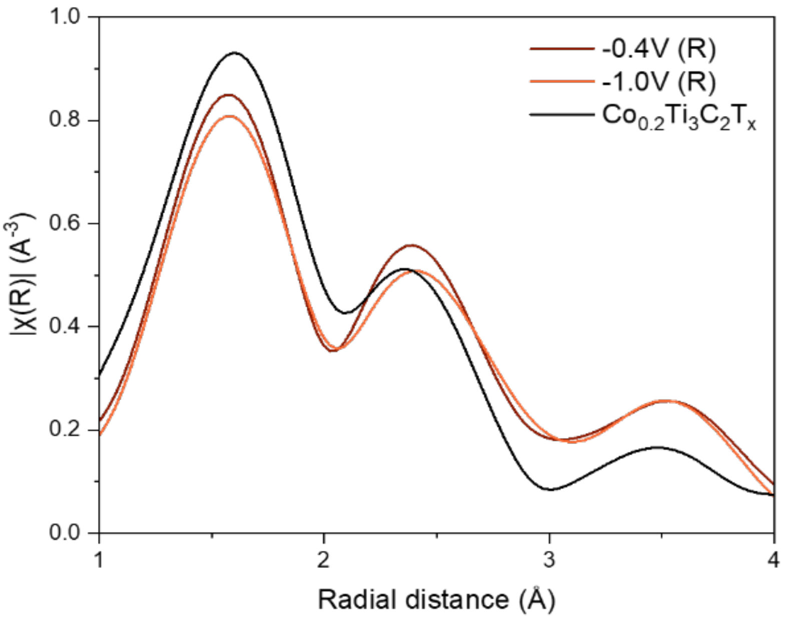

Figure S21 *The EXAFS analysis of the Co ions of Co-$Ti_3C_2T_x$ without applied potential as well as at -0.4V and -1.0V respectively.*

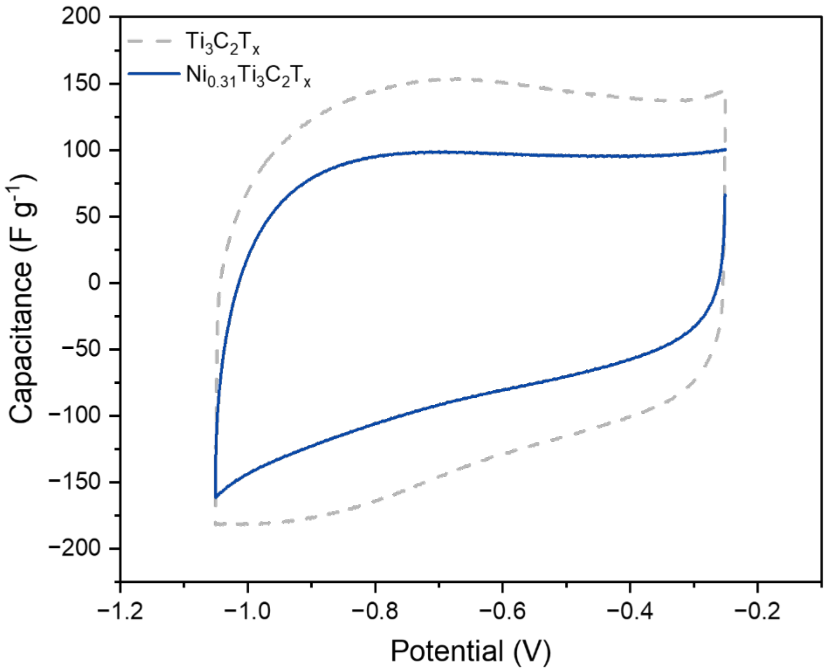

Figure S22. *The comparison of the cyclic voltammetry between $Ni_{0.31}Ti_3C_2T_x$ and $Ti_3C_2T_x$ MXenes at 1 mVs^{-1} in 0.1M NaOH, respectively.*

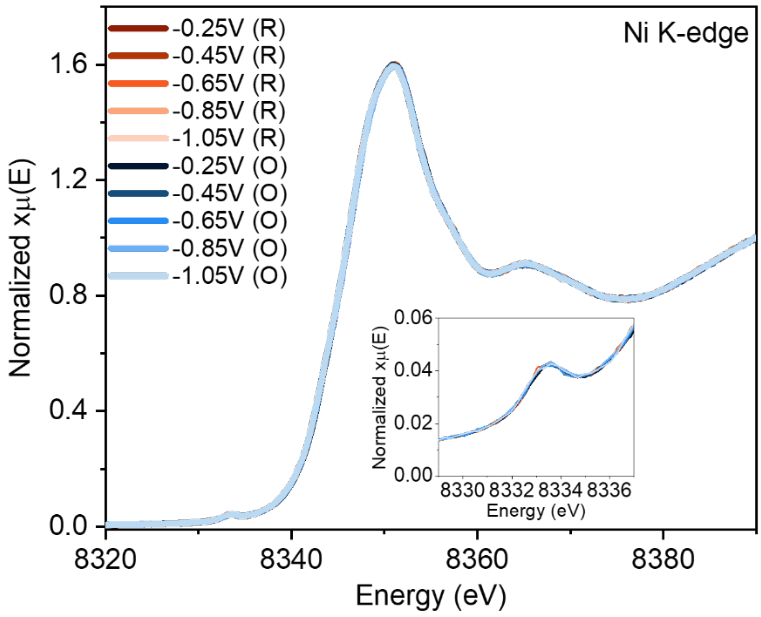

Figure S23 *The XAS of $Ni_{0.31}Ti_3C_2T_x$. The inset is the enlarged pre-edge region.*

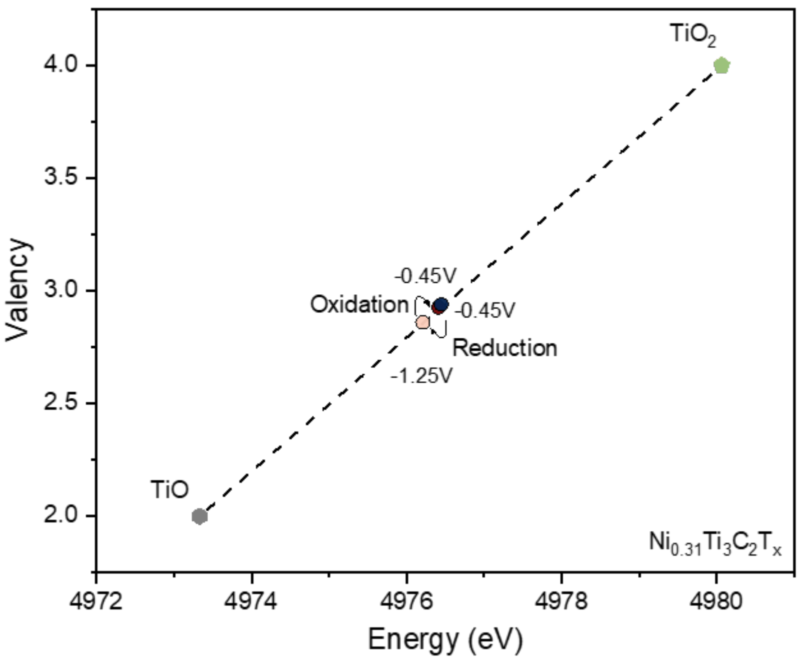

Figure S24. *Average Ti oxidation states at various potentials; Ti K-edge energies of TiO (+2) and TiO_2 (+4) are added for reference.*

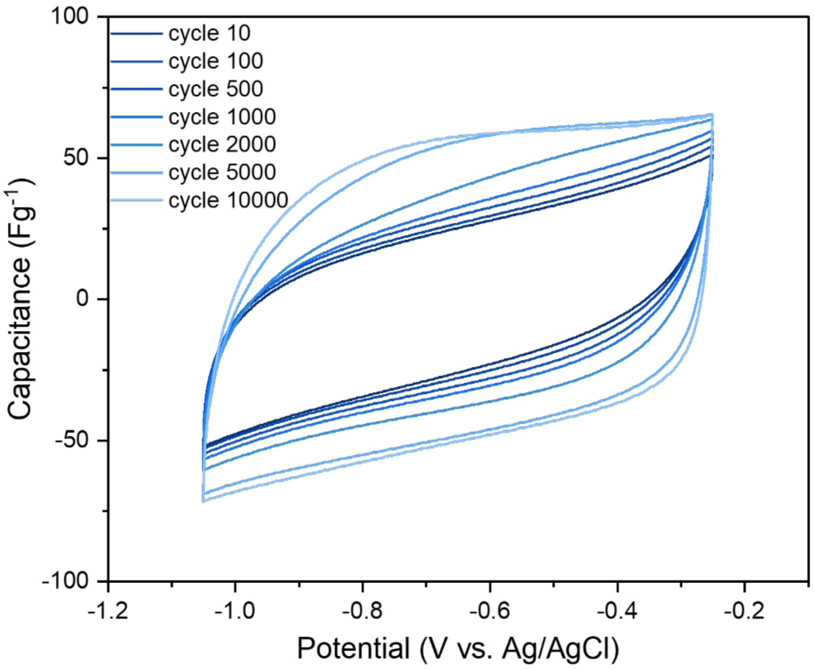

Figure S25. *The cyclic voltammetry of $Ni_{0.31}Ti_3C_2T_x$ for 10,000 cycles at $10 mVs^{-1}$.*

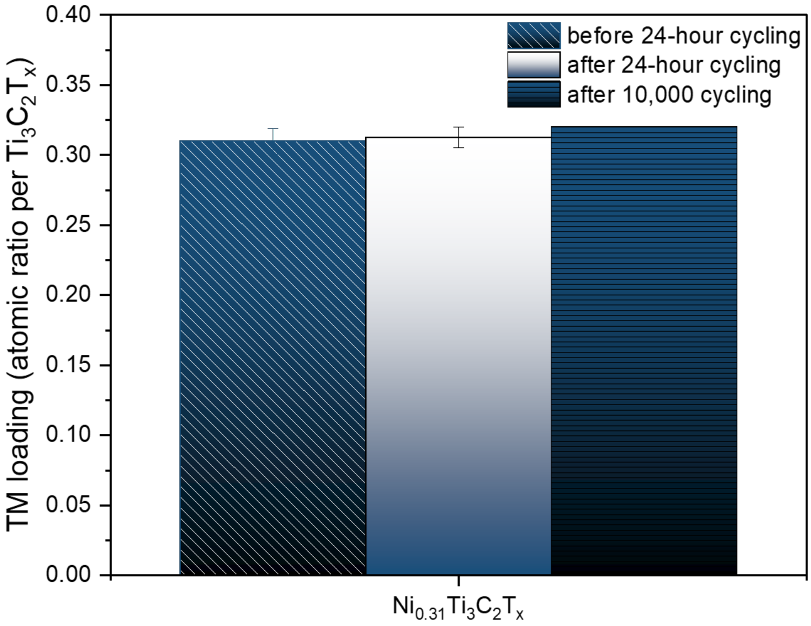

Figure S26. *The loading of Ni in $Ni_{0.31}Ti_3C_2T_x$ before and after 24-hour cycling, as well as after 10,000-cycle of cycling at 10 mVs^{-1}, quantifying through SEM-EDX.*

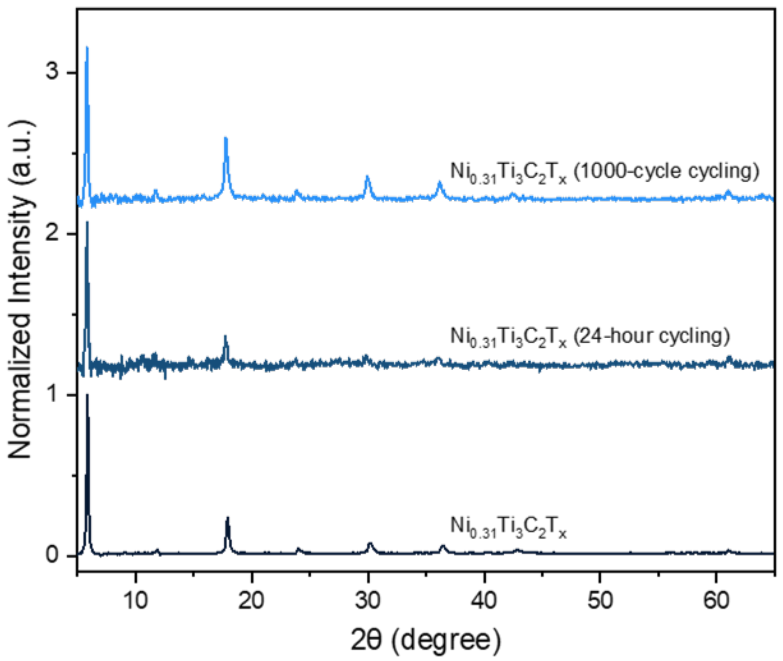

Figure S27. *XRD diffraction patterns of $Ni_{0.31}Ti_3C_2T_x$ before and after 24-hour cycling, as well as after 10,000-cycle of cycling at 10 mVs^{-1}.*

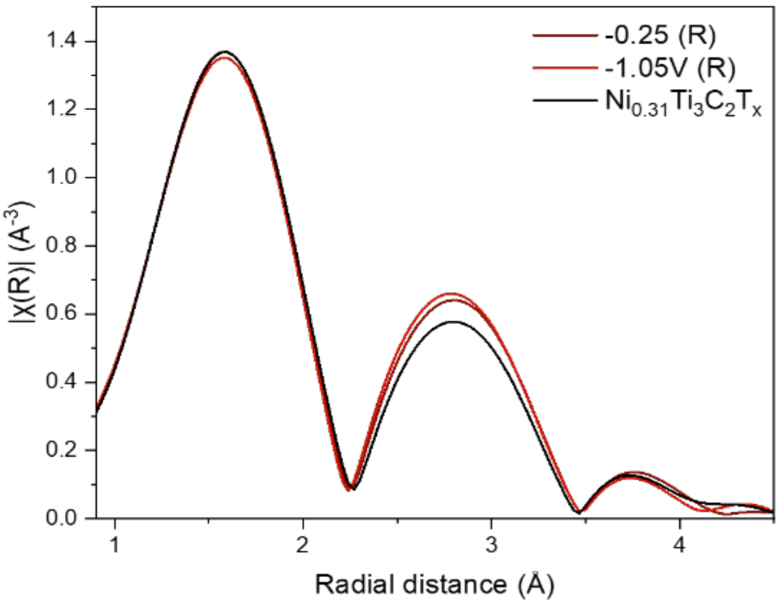

Figure S28 *The EXAFS analysis of the Ni ions Ni-$Ti_3C_2T_x$ without applied potential as well as at -0.4V and -1.0V respectively.*

Table S1. *Final composition of the simulated systems*

Composition	-O	-OH	Ratio (-OH/total)
$Ti_{27}C_{18}O_{24}H_{30}$	0	18	1
$Ti_{27}C_{18}O_{28}H_{34}Cu_2$	3	15	0.83
$Ti_{27}C_{18}O_{29}H_{36}Co_2$	4	14	0.78
$Ti_{27}C_{18}O_{29}H_{34}Ni_2$	13	5	0.28
$Ti_{27}C_{18}O_{29}H_{36}Mg_2$	3	15	0.83

Table S2. *Calculated first solvation shell distances and coordination numbers and experimental data in bulk solution.*

<table>
<tr><th>Pairs</th><th>First neighbor distance inside MXene (Å)</th><th>First neighbor distance in bulk (Å)</th><th>C.N. (MXene)</th><th>C.N. (bulk)</th></tr>
<tr><td>Cu^{2+}-O</td><td rowspan="4">1.9</td><td>2.0</td><td rowspan="2">2</td><td>5-6</td></tr>
<tr><td>Cu^{+}-O</td><td>1.8</td><td>2</td></tr>
<tr><td>Ni^{2+}-O</td><td>2.105</td><td>~3</td><td>5 or 6</td></tr>
<tr><td>Co^{2+}-O</td><td>2.08</td><td>~3.5</td><td>6</td></tr>
<tr><td>Mg^{2+}-O</td><td>2.0</td><td>2.00-2.15</td><td>4</td><td>6</td></tr>
</table>

Table S3. *Bader charges of all elements (unit: |e|).*

Element	C	Metal	H	O	Ti
$Ti_{27}C_{18}O_{24}H_{30}$	-1.6507		0.6311	-1.2920	1.5939
$Ti_{27}C_{18}O_{28}H_{34}Cu_2$	-1.6407	0.5904	0.6510	-1.2781	1.6039
$Ti_{27}C_{18}O_{29}H_{36}Co_2$	-1.6442	0.9530	0.6457	-1.2580	1.6114
$Ti_{27}C_{18}O_{29}H_{34}Ni_2$	-1.5941	0.7191	0.6632	-1.2023	1.6621
$Ti_{27}C_{18}O_{29}H_{36}Mg_2$	-1.6440	1.7583	0.6407	-1.3053	1.6084
CoO		1.1605		-1.1605	
CuO		0.9598		-0.9599	
Cu_2O		0.5252		-1.0504	
NiO		1.1360		-1.1360	
MgO		1.7248		-1.7248	
